# A Dual‐Delivery System Designed to Overcome EV‐Mediated Ferroptosis Resistance for Efficient Solid Tumor Therapy

**DOI:** 10.1002/advs.78242

**Published:** 2026-10-11

**Authors:** Yechao Shen, Zhenguo Cao, Zenan Wang, Liang Chen, Jiahao Zhang, Linjie Zeng, Boren Tan, Hengyuan Li, Jiashan Lai, Jiangchu Lei, Fanglu Chen, Peng Lin, Zhenxuan Shao, Zhan Wang

**Affiliations:** ^1^ Department of Orthopedic Surgery The Second Affiliated Hospital Zhejiang University School of Medicine Hangzhou China; ^2^ Orthopedics Research Institute of Zhejiang University Hangzhou China; ^3^ Key Laboratory of Motor System Disease Research and Precision Therapy of Zhejiang Province Hangzhou China; ^4^ Zhejiang Provincial Clinical Medical Research Center for Motor System Diseases Hangzhou China; ^5^ International Chinese Musculoskeletal Research Society Hangzhou China; ^6^ Department of Orthopedics The Second Affiliated Hospital of Xuzhou Medical University Xuzhou Jiangsu China; ^7^ Department of Pathology The Second Affiliated Hospital, Zhejiang University School of Medicine Hangzhou China

**Keywords:** ENO1, extracellular vesicles, ferroptosis, lipid nanoparticle, osteosarcoma, solid tumor

## Abstract

Tumor‐derived extracellular vesicles (EVs) are emerging as critical mediators of metabolic adaptation, yet their role in orchestrating ferroptosis resistance within the tumor microenvironment remains poorly understood. Here, we show that highly malignant osteosarcoma (OS) cells respond to ferroptotic stress by secreting EVs enriched with Alpha‐enolase (ENO1). These EVs‐ENO1 not only bolster the antioxidant capacity of tumor cells but also confer widespread ferroptosis resistance to macrophages and neutrophils, fostering an immunosuppressive niche. To disrupt this metabolic defense, we developed an OS‐targeting lipid nanoparticle (LNP) system for the synergistic delivery of BAP1 mRNA and ENO1 siRNA. We demonstrate that this dual‐delivery platform enhances ferroptosis through two complementary regulatory pathways, BAP1/SLC7A11 and ENO1/GPX4, leading to potent lipid peroxidation and tumor cell death. In syngeneic orthotopic and lung metastasis models, these targeted LNPs significantly suppress tumor progression and catalyze anti‐tumor immunity by activating M1‐type macrophages and cytotoxic T cells. Our findings identify the secretion of EVs‐ENO1 as a key reactive mechanism against ferroptosis and provide a translatable nanomedicine strategy to overcome metabolic and epigenetic resistance in solid tumors.

## Graphical abstract

(A) Establishment and characterization of the osteosarcoma‐targeted dual‐delivery nanoplatform mBAP1/siENO1@LNP‐OTP for simultaneous regulation of BAP1 and ENO1 in tumor cells. (B) After intravenous administration, mBAP1/siENO1@LNP‐OTP preferentially accumulates at tumor sites through OTP‐mediated targeting. By restoring BAP1‐mediated ferroptosis sensitivity and silencing ENO1‐mediated metabolic defense, this nanoplatform directly induces potent ferroptotic tumor cell death and suppresses the secretion of ENO1‐enriched extracellular vesicles (EVs‐ENO1), thereby blocking EV‐mediated ferroptosis resistance in neighboring tumor cells. Reduced EVs‐ENO1 further reprograms TAMs toward an M1‐like phenotype and activates cytotoxic CD8^+^ T cells, ultimately enhancing antitumor immunity.

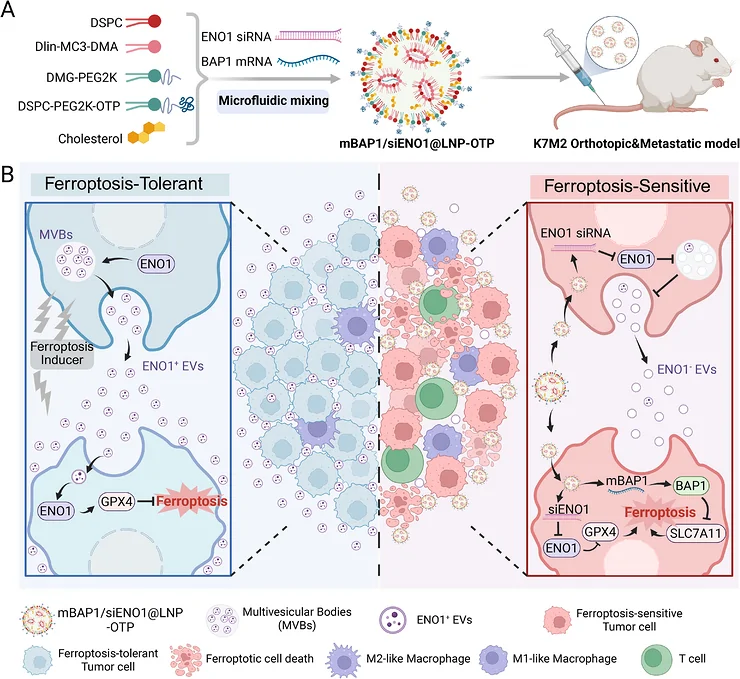

## 1 Introduction

As solid tumors represent 90% of all malignancies, adjuvant therapies like chemotherapy and immunotherapy have become increasingly vital complements to primary surgical resection [1, 2]. However, the intricate tumor microenvironment (TME), alongside inherent heterogeneity, immune evasion, and drug resistance, collectively impede the efficacy of adjuvant therapies, necessitating the urgent exploration of novel therapeutic paradigms [3, 4]. The clinical translation of Immune checkpoint blockade (ICB) remains challenging, underscored by the recent failure of various PD‐1/PD‐L1 inhibitors to demonstrate superior efficacy in Phase III trials for solid tumors [5, 6]. Notably, pivotal Phase 3 trials in small‐cell lung cancer (SCLC) have yielded suboptimal outcomes: neither the addition of ipilimumab to EP chemotherapy in 1,132 patients (NCT01450761) nor the CheckMate 451 trial investigating nivolumab plus ipilimumab (NCT02538666) succeeded in significantly prolonging patient survival relative to their respective controls [7, 8]. These setbacks underscore the urgent need to exploit alternative cell death pathways, such as ferroptosis, to overcome the limitations of current therapies and reshape the immunosuppressive landscape of solid tumors.

Ferroptosis is recognized as a metabolic pattern of cell death, serving as a natural tumor suppression mechanism that can be triggered by radiotherapy, chemotherapy, and immunotherapy [9, 10].Research indicates that drug‐resistant tumor cells exhibit marked resistance to conventional chemotherapy by evading classical apoptotic pathways, yet demonstrate particular sensitivity to ferroptosis‐inducing therapies [11, 12, 13]. Despite promising prospects, translating ferroptosis to clinical practice faces multiple challenges, including variable tumor sensitivity to ferroptosis, insufficient drug specificity, and lack of biomarkers [14, 15, 16]. Currently, tumor cells are known to evolve diverse mechanisms to resist ferroptosis, predominantly through intracellular adaptations such as the upregulation of antioxidant defense systems, the reprogramming of lipid metabolism, and the alteration of intracellular iron efflux [17, 18, 19]. Although sorafenib was initially thought to trigger iron depletion by inhibiting the xc^−^ system, this view has been challenged following more rigorous evaluations of its target specificity [20, 21]. Therefore, elucidating factors determining tumor ferroptosis sensitivity and enhancing therapeutic targeting are crucial for the application and advancement of ferroptosis‐induced antitumor strategies.

While targeting classical intracellular pathways has been extensively studied, it often ignores the collective survival networks within the TME. Extracellular vesicles (EVs) are membrane‐bound nanoparticles that mediate intercellular communication by transferring proteins, nucleic acids, and metabolites, playing a pivotal role in tumor metabolic reprogramming and immune regulation [22, 23, 24, 25]. Increasing evidence indicates EVs significantly contribute to tumor resistance to chemotherapy and immune evasion. RAB22A‐rich EVs secreted by drug‐resistant colon cancer cells induce other cancer cells in the tumor microenvironment to acquire drug resistance [26]. Further studies reveal that PD‐L1^+^ tumor‐derived EVs (TEVs) form an “EV barrier” by trapping anti‐PD‐L1 antibodies (αPD‐L1), leading to insufficient blockade of tumor PD‐L1 and subsequent resistance to αPD‐L1 therapy [27]. Emerging studies suggest that EVs can also regulate ferroptosis sensitivity in recipient cells [28, 29]. Unlike classical intracellular resistance mechanisms that act locally within single cells, TEVs facilitate the horizontal transfer of resistance factors, allowing resistant clones to confer ferroptosis resistance to neighboring sensitive cells [30, 31]. Consequently, targeting TEV‐mediated resistance offers a unique and distinct therapeutic advantage: it not only neutralizes the defense of individual cells but also dismantles the intercellular resistance network across the entire TME [32, 33]. To further elucidate the role of TEVs in the ferroptosis process of solid tumors, we examined the correlation between TEVs and tumor cell ferroptosis and confirmed that TEVs effectively enhance tumor cell ferroptosis resistance. Subsequently, we performed proteomic profiling of osteosarcoma‐derived EVs and prioritized candidate proteins based on their EV enrichment, clinical relevance, and potential involvement in ferroptosis regulation. ENO1 emerged as the most compelling candidate for subsequent mechanistic investigation. ENO1, a multifunctional glycolytic enzyme with emerging roles in tumor metabolism and stress adaptation, has recently been implicated in cancer progression and therapy resistance [34]. Through in vivo and in vitro experiments, we validated the role of EVs‐ENO1 in promoting resistance to ferroptosis‐based antitumor therapies. Therefore, developing a therapeutic system capable of precisely inhibiting ENO1 expression in TEVs represents a promising strategy to enhance the efficacy of ferroptosis‐induced antitumor treatments.

In addition to targeting ENO1‐driven ferroptosis resistance, we selected BAP1 mRNA as a therapeutic cargo due to its central role as an upstream epigenetic regulator of the SLC7A11 antioxidant system [35, 36]. BAP1‐mediated transcriptional repression of SLC7A11 provides a sustained strategy to enhance ferroptosis sensitivity at the transcriptional level, complementing the post‐transcriptional inhibition of ENO1.

RNA therapeutics enable precise gene regulation and have been widely applied to various refractory diseases [37, 38]. Small interfering RNA (siRNA) selectively silences oncogenes, while messenger RNA (mRNA) encodes tumor suppressor genes and reprograms immune responses [39, 40]. Combining these modalities achieves synergistic regulation, amplifying therapeutic effects. Lipid nanoparticles (LNPs) represent a leading non‐viral delivery platform for siRNA or mRNA [39, 41]. In recent years, combined delivery strategies have garnered increasing attention and demonstrated considerable therapeutic potential [42, 43]. However, delivering LNPs to tumor sites beyond the liver remains a significant challenge due to their non‐targeted effects and instability in blood and organ tissues [44]. In recent years, our laboratory has focused on developing and applying targeted biomaterials for precision cancer therapy, including functional peptides, nanoparticles, and engineered delivery systems [45, 46, 47]. Therefore, in this study, we employed a modification strategy based on tumor‐targeting peptides screened from phage display to enhance the therapeutic selectivity of LNPs toward tumors.

In this study, we discovered that the key molecule ENO1 in TEVs enhances tumor resistance to ferroptosis. Consequently, we designed an osteosarcoma‐targeting peptide (OTP)‐modified lipid nanoparticle co‐delivery system, termed mBAP1/siENO1@LNP‐OTP, to simultaneously deliver BAP1 mRNA and ENO1 siRNA, thereby inducing ferroptosis in tumor cells and overcoming ferroptosis resistance. In this system, LNPs serve as RNA carriers, while the surface‐modified OTP enables tumor‐targeted delivery of both therapeutic cargos. This targeted co‐delivery strategy effectively silenced the ENO1 gene in tumor cells, leading to a significant reduction in EVs‐ENO1 and restoring the tumor suppressor gene BAP1 (which induces ferroptosis). This induced redox imbalance and lipid peroxidation, significantly inhibiting tumor growth. Collectively, this study proposes a therapeutic strategy targeting EVs‐ENO1 to reduce tumor ferroptosis resistance, aiming to enhance ferroptosis sensitivity and provide potential targets and strategies for precision treatment of related tumors. A schematic overview of the study design and proposed mechanism is presented in Scheme 1.

**SCHEME 1:**
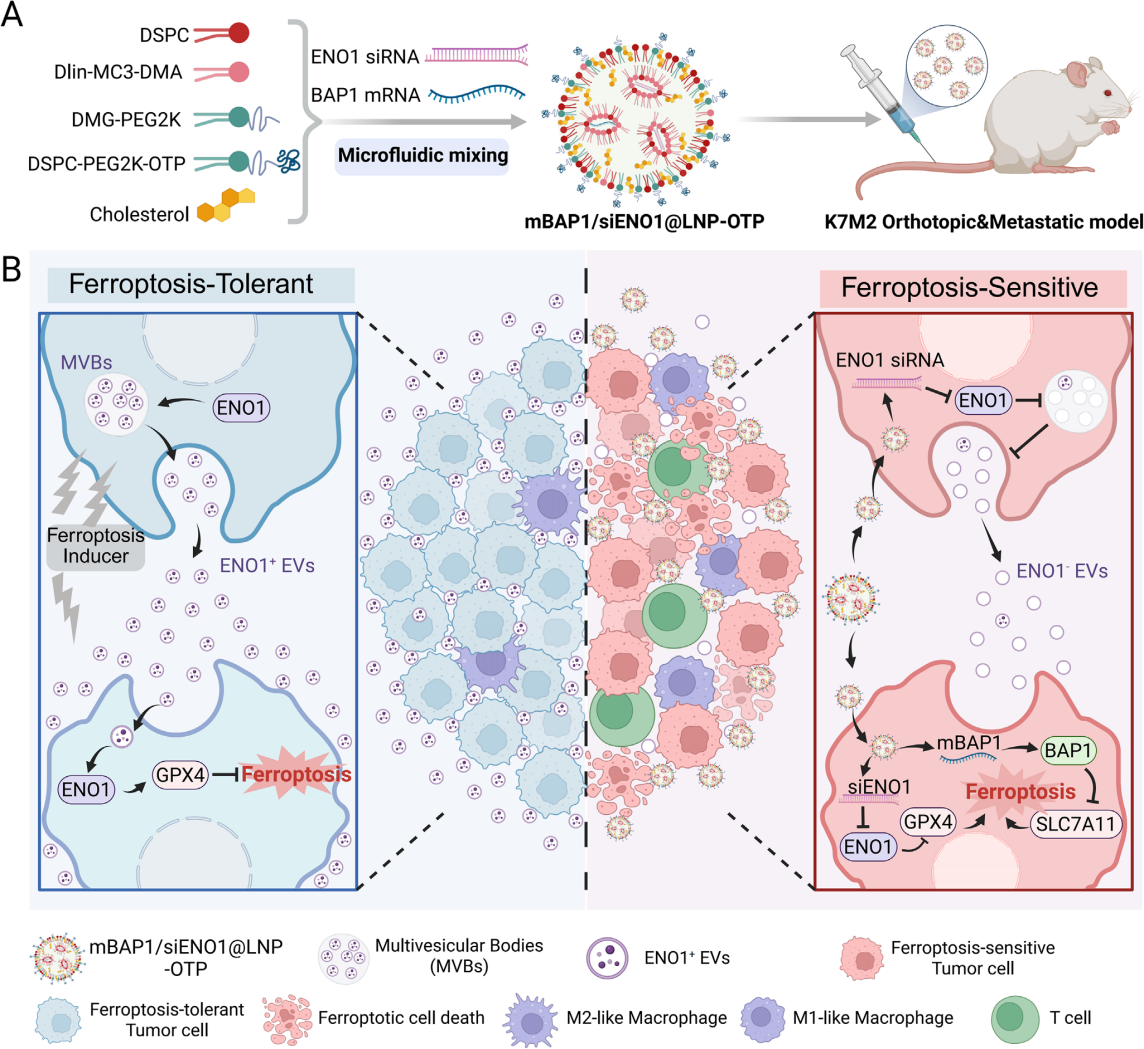
Schematic illustration of an OS‐targeted LNP system for coordinated regulation of the BAP1/SLC7A11 and ENO1/GPX4 pathways to dismantle EV‐mediated ferroptosis resistance. (A) Establishment and characterization of a dual‐delivery system (mBAP1/siENO1@LNP‐OTP) for regulating BAP1 and ENO1 in tumor cells. (B) mBAP1/siENO1@LNP‐OTP accumulated in the tumor site through OTP‐mediated tumor targeting after i.v. administration, and directly triggered potent tumor cell ferroptosis by restoring BAP1‐mediated epigenetic sensitivity and silencing ENO1‐mediated metabolic defense. This intervention effectively inhibited the secretion of ENO1‐enriched extracellular vesicles (EVs‐ENO1), which otherwise confers ferroptosis resistance to neighboring cells. Consequently, the reduction of EVs‐ENO1 reprogrammed the TAM phenotype toward M1 and activated cytotoxic CD8^+^ T cells, thereby enhancing anti‐tumor immunity.

## 2 Results

### 2.1 Tumor‐Derived EVs Sequester ENO1 to Establish a Reactive Defense Against Ferroptosis

To investigate whether EVs contribute to tumor ferroptosis resistance, we first examined the relationship between ferroptotic stress and EVs secretion (Figure 1A). Initially, cells were treated with various concentrations of RSL3 for 24 h. We identified 0.5 µM as the “safe dose,” defined as the maximum concentration that does not impair tumor cell proliferation (Figure S1A). Under conditions that did not compromise cell viability, we collected EVs secreted by an equal number of tumor cells following treatment with different concentrations of FINs (the ferroptosis inducer RSL3). To avoid ambiguity in vesicle nomenclature, we used the term tumor‐derived extracellular vesicles (TEVs) throughout the revised manuscript to describe the vesicle‐enriched population isolated from tumor cell‐conditioned medium. The isolated TEVs were comprehensively characterized before subsequent functional analyses. Transmission electron microscopy (TEM) revealed a typical cup‐shaped vesicular morphology (Figure S1B). Nanoparticle tracking analysis (NTA) showed that the isolated vesicles displayed a relatively homogeneous size distribution, predominantly ranging from 100 to 200 nm, with a particle concentration of 8.01 × 10^10^ ± 1.15 × 10^10^ particles/mL (Figure S1C). Western blot analysis further confirmed the enrichment of canonical EV markers, including CD9 and TSG101, whereas the intracellular/endoplasmic reticulum protein Calnexin was undetectable, supporting the EV identity of the isolated vesicles and indicating minimal cellular contamination (Figure S1D). The zeta potential of the isolated TEVs was approximately −14.2 mV (Figure S1E). We observed that FINs significantly increased the number of TEVs in the culture supernatant of K7M2 osteosarcoma (OS) cells (Figure 1B). Similar results were observed in other highly malignant cell lines, such as 4T1 (breast cancer) and LLC (lung cancer), suggesting that increasing TEVs secretion is a reactive response of tumor cells to ferroptotic stress (Figure S1F,G).

**FIGURE 1:**
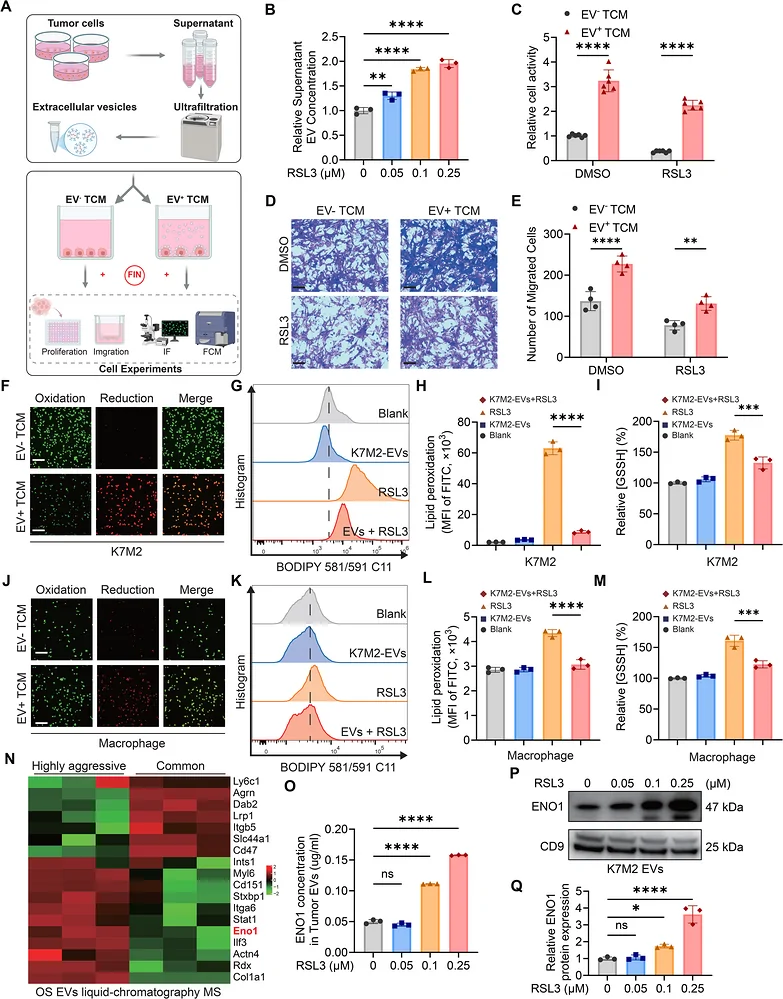
Tumor‐derived EVs inhibit ferroptosis and promote progression in K7M2 cells and macrophages. A: Schematic workflow of the experimental design (created with BioRender.com). TEVs were isolated from tumor cell culture supernatants via ultracentrifugation. Recipient cells were treated with ferroptosis inducers in the presence or absence of TEVs at a final concentration of 10 µg/mL EV protein, followed by various functional assays. B: Quantification of TEV secretion from K7M2 cells following treatment with different concentrations of RSL3, rather than ENO1 levels in TEVs. C: CCK‐8 assay showing the viability of K7M2 cells treated with medium containing or lacking TEVs at a final concentration of 10 µg/mL EV protein (n = 5). D–E: Representative images (D) and quantification (E) of Transwell assays for K7M2 cells stimulated with the ferroptosis inducer RSL3 in the presence or absence of TEVs at a final concentration of 10 µg/mL EV protein (n = 4). F: Representative confocal images of lipid peroxidation in K7M2 cells treated with or without TEVs (10 µg/mL EV protein) and stained with BODIPY 581/591 C11. Green fluorescence indicates oxidized BODIPY C11, and red fluorescence indicates reduced BODIPY C11. G–H: Representative flow cytometry plots (G) and quantification (H) of lipid peroxidation changes in K7M2 cells treated with or without TEVs (10 µg/mL EV protein) using BODIPY 581/591 C11 staining (n = 3). I: Relative levels of oxidized glutathione disulfide (GSSG) in K7M2 cells treated with or without TEVs (10 µg/mL EV protein) under the indicated conditions (n = 3). J: Representative images of lipid peroxidation in macrophages treated with or without TEVs (10 µg/mL EV protein) and stained with BODIPY 581/591 C11. Green fluorescence indicates oxidized BODIPY C11, and red fluorescence indicates reduced BODIPY C11. K–L: Representative flow cytometry plots (K) and quantification (L) of lipid peroxidation changes in macrophages treated with or without TEVs (10 µg/mL EV protein) under the indicated conditions (n = 3). M: Relative GSSG levels in macrophages treated with or without TEVs (10 µg/mL EV protein) under the indicated conditions (n = 3). N: Heatmap displaying differentially expressed protein cargos in K7M2‐EVs compared with K7‐EVs, as identified by LC‐MS/MS (n = 3). O: ENO1 levels in TEVs from K7M2 cells treated with varying concentrations of RSL3, measured by ELISA (n = 3). P–Q: Representative Western blot (P) and densitometric quantification (Q) of ENO1 expression in TEVs derived from K7M2 cells treated with the indicated concentrations of RSL3 (n = 3). Data are presented as mean ± s.e.m. Statistical significance was determined by unpaired two‐tailed Student's t‐test for two‐group comparisons, one‐way ANOVA followed by Tukey's post hoc test for multiple‐group comparisons, and two‐way ANOVA for time‐course analyses. ns, not significant; *p < 0.05, **p < 0.01, ***p < 0.001, ****p < 0.0001.

To further verify whether these TEVs are associated with the regulation of ferroptosis resistance, we performed flow cytometry and immunofluorescence assays in vitro (Figure 1A). Given our focus on OS, the K7M2 cell line was used as the primary experimental model. Our results showed that TEVs treatment initially promoted the proliferation of K7M2 cells; notably, after the addition of RSL3, TEVs effectively preserved the proliferative activity of K7M2 cells and mitigated the inhibitory effects of ferroptosis (Figure 1C). Concurrently, TEVs effectively counteracted FIN‐mediated inhibition of tumor cell migration (Figure 1D,E). We then assessed lipid peroxidation levels in RSL3‐treated K7M2 cells. Fluorescence imaging indicated that TEVs treatment significantly reduced FIN‐induced lipid peroxidation, maintaining cells in a more reductive state (Figure 1H, Figure S1I). Flow cytometry confirmed that while FIN treatment significantly upregulated lipid peroxidation in K7M2 cells, the addition of TEVs effectively reversed this effect (Figure 1G,H). Intracellular glutathione assays revealed that the proportion of oxidized glutathione (GSSG) was significantly reduced by TEVs, while the proportion of reduced glutathione (GSH) increased (Figure 1I, Figure S1J). These findings suggest that TEVs enhance the ferroptosis tolerance of tumor cells.

As TEVs are distributed throughout the TME in vivo, we further examined their regulatory effects on macrophages and neutrophils. Mouse bone marrow‐derived macrophages (BMDMs) and neutrophils were successfully obtained through bone marrow extraction. Fluorescence and flow cytometry results showed that K7M2‐EVs similarly and significantly reduced lipid peroxidation levels in macrophages and neutrophils, maintaining them in a more reductive state (Figure 1J–L, Figure S1L,M). Glutathione assays demonstrated that the intracellular GSSG proportion in macrophages and neutrophils treated with TEVs was also significantly reduced (Figure 1 M, Figure S1K, S1N,O). This indicates that the anti‐ferroptotic effect of TEVs is widespread, influencing various cells within the TME.

To identify potential functional molecules within TEVs responsible for regulating ferroptosis resistance, we analyzed differential TEV cargo proteins between malignant and conventional OS cells using a publicly available mass spectrometry dataset [48]. Functional annotation of the identified EV proteins revealed enrichment of pathways associated with metabolic regulation and cellular stress responses (Figure S2A,B). Since ferroptosis is closely linked to metabolic reprogramming and redox homeostasis, we further prioritized candidates based on their biological relevance, clinical significance, and potential involvement in ferroptosis regulation. Among these candidates, ENO1 was selected for further investigation because it was significantly enriched in malignant OS‐derived EVs (Figure 1N) and has been implicated in regulating lipid peroxide accumulation and ferroptosis resistance in other tumor contexts [49]. Therefore, we hypothesized that EV‐associated ENO1 may represent a key mediator of tumor resistance against ferroptosis.

Based on pan‐cancer analysis of the TCGA database and TARGET‐OS tumor tissue sequencing, we found that ENO1 is significantly upregulated in OS tissues (Figure S2C,D). Furthermore, analysis using TARGET‐OS and the GSE237033 dataset indicated that ENO1 is significantly upregulated in OS patients with metastasis, suggesting a correlation with poor clinical progression (Figure S2E,F).

To further examine the clinical relevance of ENO1 in OS, we analyzed primary tumor tissues from a total of 184 OS patients enrolled in our hospital cohort, including both metastatic (n = 98) and non‐metastatic (n = 86) cases (Table S4). ENO1 expression was assessed by immunohistochemical (IHC) staining. The results demonstrated that ENO1 expression was significantly upregulated in tumor tissues from metastatic patients compared with non‐metastatic patients (Figure S2G). This trend was consistently observed across the entire cohort, supporting a robust association between elevated ENO1 expression and metastatic disease status in OS. Prognostic analysis also showed that patients in TARGET‐OS with high ENO1 expression have shorter survival times (Figure S2H). These results suggest that ENO1 plays a vital role in OS progression.

To explore changes in ENO1 within TEVs, we measured the concentration of EVs‐ENO1 following FIN treatment. ELISA results showed that K7M2‐derived EVs‐ENO1 secretion increased in a dose‐dependent manner following FIN stimulation (Figure 1O). Western Blotting (WB) experiments further validated this phenomenon (Figure 1P,Q). Additionally, we observed that EVs‐ENO1 levels in breast and lung cancer cells also significantly increased upon FIN stimulation (Figure S2I,J). In summary, our results indicate that tumor‐secreted EVs are a key regulatory mechanism inducing ferroptosis tolerance, with EVs‐ENO1 likely serving as a potential critical molecule.

### 2.2 Vesicle‐Associated ENO1 Facilitates Tumor Progression and Lung Metastasis by Bolstering Ferroptotic Resistance

To further explore the role of EVs‐ENO1 in tumor ferroptosis, we first constructed a K7M2 cell line carrying Flag‐tagged ENO1 (Figure S3C). Immunofluorescence results showed that EVs‐ENO1 in the OS supernatant effectively bound to tumor cells (Figure 2A).

**FIGURE 2:**
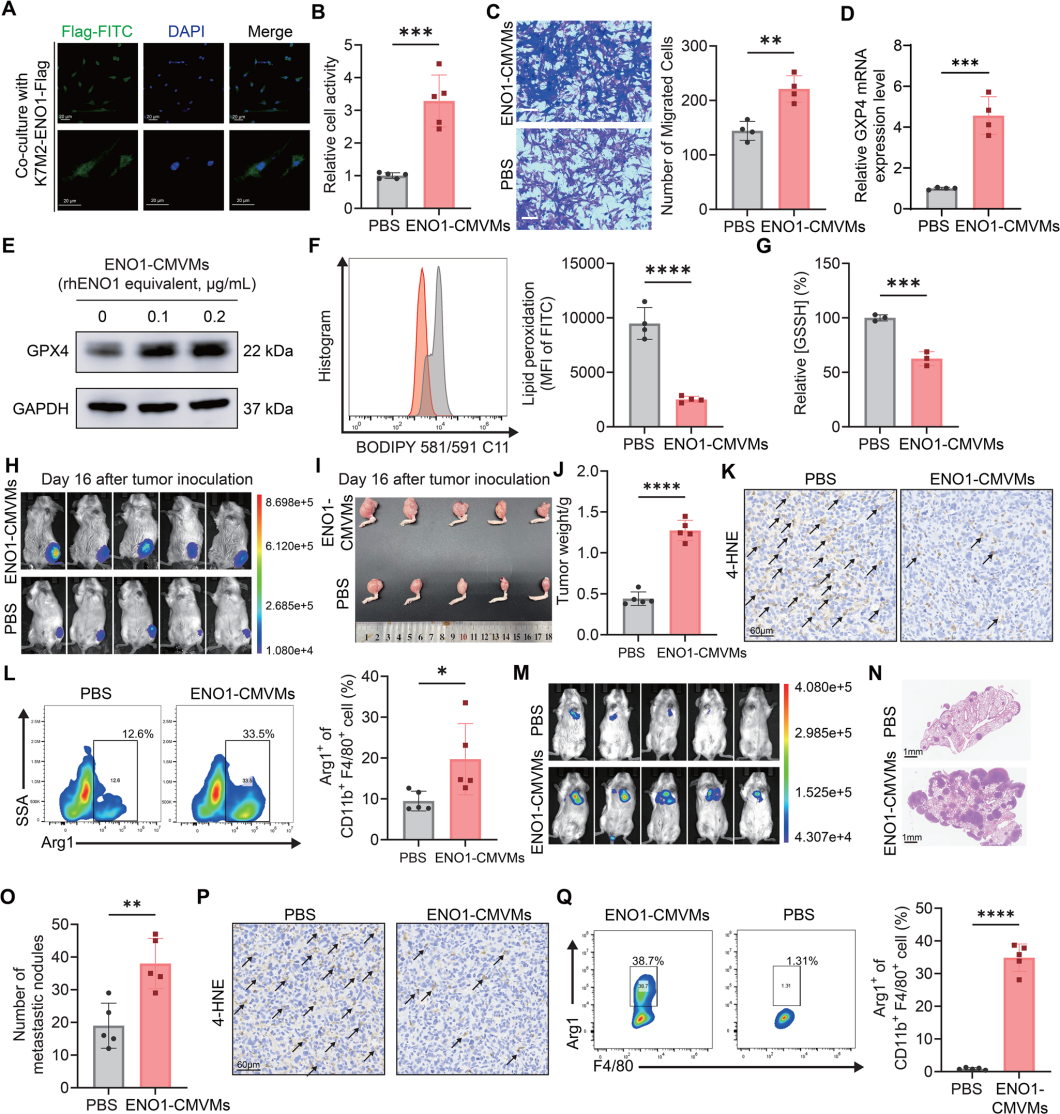
ENO1‐CMVMs promote ferroptosis resistance and tumor progression in vivo and in vitro. A: Representative IF images showing the uptake of Flag‐labeled ENO1 by K7M2 tumor cells. Nuclei were stained with DAPI. Scale bar: 20 µm. B: Cell viability assay. Relative cell viability of K7M2 cells treated with or without ENO1‐CMVMs under RSL3‐induced ferroptosis stress (n = 5). C: Cell migration assay. Representative images and statistical analysis of Transwell assays for K7M2 cells treated with or without ENO1‐CMVMs under RSL3 stimulation (n = 4). D: GPX4 mRNA expression analysis. RT‐qPCR analysis of GPX4 mRNA expression in K7M2 cells treated with PBS or ENO1‐CMVMs (n = 4). E: Western blotting (WB) assay. WB analysis of GPX4 protein expression in K7M2 cells treated with ENO1‐CMVMs. For ENO1‐CMVM treatments, the indicated concentrations refer to the amount of encapsulated rhENO1 (rhENO1‐equivalent concentration), rather than the total CMVM protein concentration. F: Lipid peroxidation analysis. Representative flow cytometry plots and quantification of lipid peroxidation changes in K7M2 cells treated with or without ENO1‐CMVMs using BODIPY 581/591 C11 staining (n = 4). G: Oxidative stress marker. Relative GSSG levels in K7M2 cells treated with or without ENO1‐CMVMs (n = 3). H: In vivo bioluminescence imaging. Representative bioluminescence images and quantitative analysis of intratibial tumors in mice (day 16) treated with ENO1‐CMVMs (50 µg) or control (n = 5 per group). I–J: Tumor morphology and weight. Representative photographs (I) and weight measurements (J) of intratibial tumors harvested from mice treated with ENO1‐CMVMs or PBS (n = 5). K: Immunohistochemistry (IHC) for 4‐HNE. Representative IHC staining images of 4‐HNE (a marker of lipid peroxidation) in tumor tissues. Scale bar: 60 µm. L: Macrophage polarization (Primary tumor). Representative flow cytometry plots and statistical analysis of Arginase 1 (Arg1) expression in CD11b^+^ F4/80^+^ macrophages harvested from K7M2 intratibial tumors on day 16 (n = 5). M: Lung metastasis imaging. Bioluminescence images of lung metastatic burden in mice treated with ENO1‐CMVMs or PBS on day 14 (n = 5 per group). N–O: Lung nodule assessment. Representative H&E‐stained lung sections (N) and quantification of metastatic nodules (O) (n = 5). P: IHC for 4‐HNE (Lung). Representative 4‐HNE staining in lung metastatic lesions. Scale bar: 60 µm. Q: Macrophage polarization (Lung). Flow cytometric analysis and quantification of Arginase 1 (Arg1) expression in CD11b^+^ F4/80^+^ macrophages within lung metastatic tumors on day 14 (n = 5). Data are presented as mean ± s.e.m. Statistical significance was determined by unpaired two‐tailed Student's t‐test for two‐group comparisons, one‐way ANOVA followed by Tukey's post hoc test for multiple‐group comparisons, and two‐way ANOVA for time‐course analyses. ns, not significant; *p < 0.05, **p < 0.01, ***p < 0.001, ****p < 0.0001.

To examine the contribution of vesicle‐associated ENO1 in a simplified and controlled setting, we established a reductionist biomimetic system. Because recombinant human ENO1 (rhENO1) was used as the protein cargo to generate ENO1‐loaded cell membrane‐derived vesicle mimetics (ENO1‐CMVMs) for treatment of murine K7M2 cells, we first performed sequence alignment between human and mouse ENO1 proteins to evaluate cross‐species conservation. Human and mouse ENO1 shared more than 99.5% amino acid sequence identity, with complete conservation of the catalytic and substrate‐binding residues, supporting the high functional conservation of ENO1 between these two species (Supplementary Information S1).

Specifically, rhENO1 was incorporated into CMVMs using an extrusion‐based method to generate ENO1‐CMVMs, thereby simulating EV‐associated ENO1 delivery in a controlled manner. K7M2 cell membrane‐derived vesicle mimetics were co‐extruded with rhENO1, followed by ultrafiltration to remove free rhENO1. The resulting ENO1‐CMVMs displayed nanoscale vesicle‐like morphology, an average hydrodynamic diameter of approximately 145 nm, a zeta potential of −6.4 mV, and an rhENO1 encapsulation efficiency of approximately 42.5% (Figure S3D–G). This system enabled selective interrogation of ENO1 function while minimizing potential confounding effects from other endogenous EV components. Using this system, we found that ENO1‐CMVMs significantly preserved the proliferative and migratory capacities of K7M2 cells under ferroptotic stress induced by FINs (Figure 2B,C). Mechanistically, ENO1‐CMVMs markedly upregulated GPX4, a central negative regulator of ferroptosis (Figure 2D,E). Consistently, ENO1‐CMVMs effectively reduced intracellular lipid peroxidation and the GSSG proportion in K7M2 cells, further supporting the anti‐ferroptotic role of EV‐associated ENO1 (Figure 2F,G).

To further verify the role of EVs‐ENO1 in antagonizing ferroptosis in vivo, we examined its effects in mice. We established a mouse orthotopic tibial tumor model and administered ENO1‐CMVMs via tail vein injection every three days (50 µg). Although FIN treatment (5 mg/kg, i.p.) significantly inhibited tumor growth, systemic injection of ENO1‐CMVMs allowed tumor progression to recover, resulting in tumor volumes significantly larger than those in the PBS treatment group (Figure 2H–J). Immunohistochemical (IHC) staining for 4‐hydroxynonenal (4‐HNE), a marker of ferroptosis, confirmed that ENO1‐CMVMs effectively inhibited ferroptosis within the tumor tissue (Figure 2K). Simultaneously, we investigated the remodeling of the TME following ENO1‐CMVMs treatment. Flow cytometry analysis showed a significant increase in M2‐like macrophages (Arg1^+^) and a decrease in M1‐like macrophage (iNOS^+^) infiltration in the ENO1‐CMVMs treatment group, indicating an intensified immunosuppressive TME (Figure 2L, Figure S3H). To explore its impact on distant metastasis, we employed a tail vein injection‐induced lung metastasis model. The results demonstrated that ENO1‐CMVMs significantly promoted K7M2 lung metastasis, with a markedly higher number of metastatic nodules compared to the PBS group (Figure 2M–O). IHC analysis further confirmed that ferroptosis was similarly inhibited by ENO1‐CMVMs at the metastatic sites, and flow cytometry revealed decreased immune cell activation levels (Figure 2P). Flow cytometry also identified immunosuppressive phenomena at the lung metastasis sites (Figure 2Q, Figure S3I). In conclusion, these findings demonstrate that ENO1‐CMVMs effectively counteract the anti‐tumor effects of FINs in vivo, further supporting vesicle‐associated ENO1 as a promising therapeutic target for ferroptosis‐based interventions.

### 2.3 Engineering OTP‐Functionalized LNPs for Precision Co‐Delivery of BAP1 mRNA and ENO1 siRNA

To counteract EVs‐ENO1‐mediated ferroptosis resistance, we developed a multifunctional LNP platform for tumor‐targeted co‐delivery of BAP1 mRNA and ENO1 siRNA (Figure 3A). The LNPs were formulated using a microfluidic mixing approach with an optimized lipid composition consisting of DLin‐MC3‐DMA, DSPC, cholesterol, DMG‐PEG2K, and DSPE‐PEG2K‐OTP (50:10:38.5:1.0:0.5 mol%), in which the total PEG‐lipid content was maintained at 1.5 mol%. The incorporation of DSPE‐PEG2K‐OTP enabled surface presentation of the osteosarcoma‐targeting peptide (OTP), while DMG‐PEG2K was included to maintain colloidal stability and facilitate PEG shedding after systemic administration.

**FIGURE 3:**
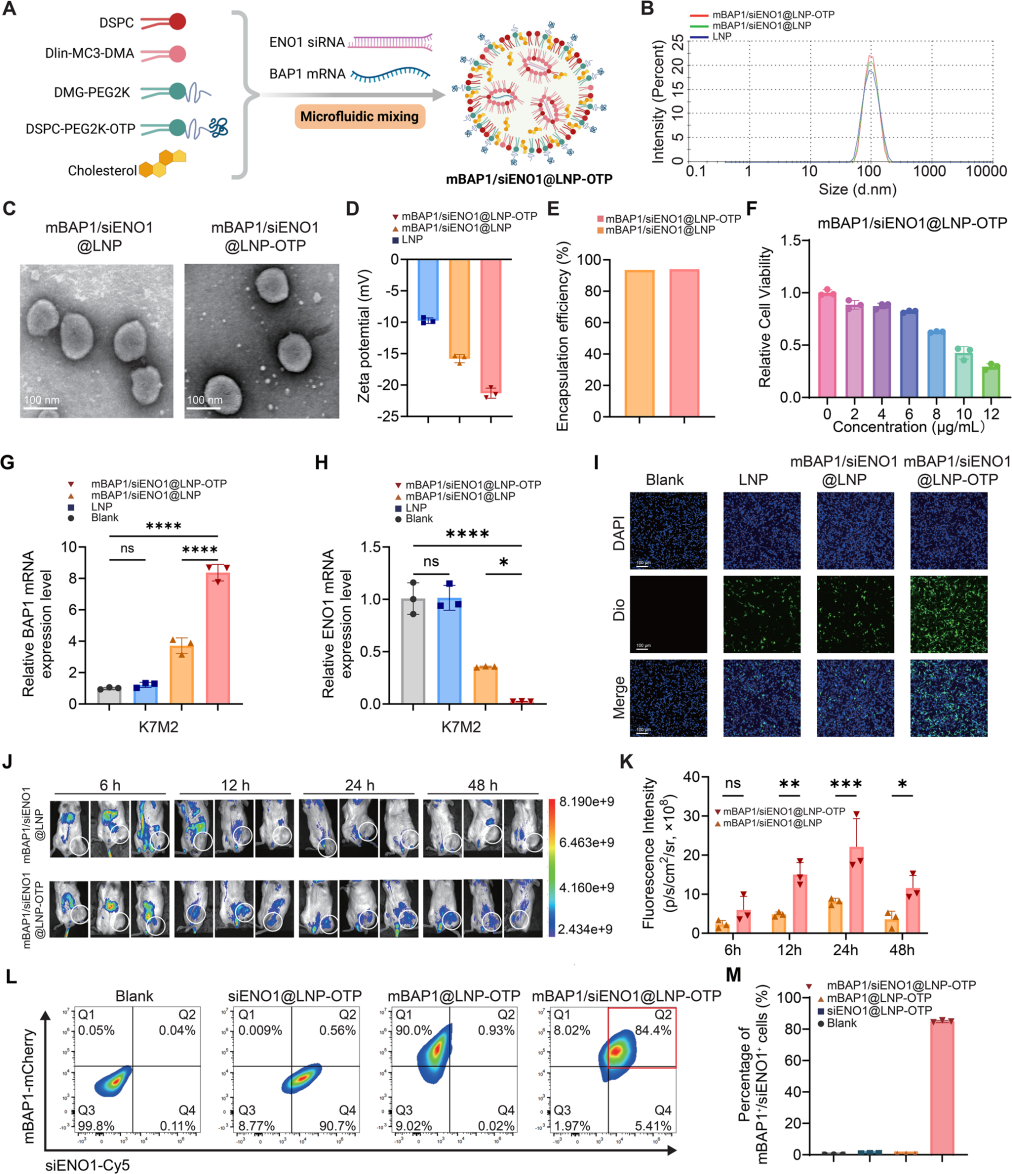
Synthesis and characterization of the targeted delivery system. A: Schematic of nanoparticle fabrication. Illustrative workflow showing the synthesis and assembly of the delivery system. B: Particle size distribution. Hydrodynamic diameters of LNP, mBAP1/siENO1@LNP, and mBAP1/siENO1@LNP‐OTP as determined by Dynamic Light Scattering (DLS) analysis. C: Morphological characterization. Representative Transmission Electron Microscopy (TEM) images of the nanoparticles. Scale bar: 100 nm. D: Surface charge. Zeta potential values of the various nanoparticle formulations. E: Encapsulation efficiency. Analysis of the loading and encapsulation efficiency of the therapeutic cargos within the nanoparticles. F: In vitro cytotoxicity. Cell viability of K7M2 treated with mBAP1/siENO1@LNP‐OTP, evaluated via CCK‐8 assay (n = 3). G: BAP1 mRNA expression. RT‐qPCR analysis of BAP1 mRNA levels in K7M2 cells following treatment with PBS, LNP, mBAP1/siENO1@LNP, or mBAP1/siENO1@LNP‐OTP for 24 h (n = 3). H: ENO1 mRNA expression. RT‐qPCR analysis of ENO1 mRNA levels in K7M2 cells following the indicated treatments for 24 h (n = 3). I: Cellular uptake and internalization. Representative fluorescence images of DiO‐labeled LNP, mBAP1/siENO1@LNP, and mBAP1/siENO1@LNP‐OTP internalized by K7M2 cells. Scale bar: 100 µm. J‐K: In vivo fluorescence imaging showed targeted delivery of mBAP1/siENO1@LNP, and mBAP1/siENO1@LNP‐OTP in intratibial tumor sites at the indicated time points (J) and Fluorescence intensity in the intratibial tumor sites (n = 3). L‐M: Representative flow cytometry plots (L) and quantification (M) of mBAP1‐mCherry^+^/siENO1‐Cy5^+^ double‐positive K7M2 cells after treatment with blank LNP‐OTP, siENO1@LNP‐OTP, mBAP1@LNP‐OTP, or mBAP1/siENO1@LNP‐OTP (n = 3). Data are presented as mean ± s.e.m. Statistical significance was determined by unpaired two‐tailed Student's t‐test for two‐group comparisons, one‐way ANOVA followed by Tukey's post hoc test for multiple‐group comparisons, and two‐way ANOVA for time‐course analyses. ns, not significant; *p < 0.05, **p < 0.01, ***p < 0.001, ****p < 0.0001.

The synthesized LNPs efficiently encapsulated BAP1 mRNA and ENO1 siRNA (Figure S4A). The OTP‐conjugated lipid‐peptide (QYWNIQNGPYGG‐KKK‐K(pal)) was synthesized and characterized by high‐performance liquid chromatography (HPLC) and electrospray ionization mass spectrometry (ESI‐MS). As shown in Figure S4B, the HPLC chromatogram exhibited a single dominant peak with a purity of 91.03%, and the molecular weight determined by ESI‐MS was 2147.56 Da, consistent with the theoretical molecular mass of the designed peptide. These results confirmed the successful synthesis of high‐purity OTP‐conjugated lipid components.

Next, we evaluated the characteristics of mBAP1/siENO1@LNP‐OTP and other nanovesicles. Dynamic Light Scattering (DLS) analysis showed that mBAP1/siENO1@LNP and mBAP1/siENO1@LNP‐OTP had similar sizes, exhibiting a uniform spherical morphology with an average diameter of approximately 94 nm and a narrow polydispersity index (PDI) (Figure 3B, Figure S4A). Transmission Electron Microscopy (TEM) analysis revealed that both LNP samples primarily consisted of particles with electron‐dense cores (Figure 3C). Both mBAP1/siENO1@LNP and mBAP1/siENO1@LNP‐OTP exhibited negative zeta potentials (Figure 3D). Compared to mBAP1/siENO1@LNP, the OTP‐modified version showed an increased negative zeta potential, confirming successful surface modification (Figure 3D). The encapsulation efficiency for both mRNA and siRNA exceeded 90%, ensuring high drug loading (Figure 3E). In vitro cytotoxicity was assessed using the CCK8 assay by incubating K7M2 cells with different concentrations of nanoparticles for 24 h. At a concentration of 4 µg/mL, mBAP1/siENO1@LNP‐OTP began to exhibit slight cytotoxicity (cell viability ∼92%), while concentrations exceeding 10 µg/mL resulted in significant cytotoxicity (cell viability <50%) (Figure 3F). Similar trends were observed for mBAP1/siENO1@LNP (Figure S4C). These results indicate the successful synthesis of lipid nanoparticles targeting both ENO1 and BAP1.

Targeting specificity and transport efficiency are crucial for siRNA and mRNA transfection. We subsequently assessed the targeting specificity of the OTP modification and the silencing/overexpression efficacy of the dual‐delivery system. qPCR results showed that mBAP1/siENO1@LNP‐OTP exhibited the strongest BAP1 upregulation and ENO1 downregulation compared to other groups (Figure 3G,H). In vitro uptake experiments using fluorescence‐labeled LNPs showed that K7M2 cells took up significantly more LNP‐OTP than non‐targeted LNPs (Figure 3I, Figure S4D). To rigorously confirm that this enhanced internalization is specifically mediated by OTP‐receptor recognition, a competitive inhibition assay was conducted. K7M2 cells were pre‐incubated with an excess of free OTP peptide prior to treatment with the targeted LNPs. This receptor‐blocking pre‐treatment significantly reduced the cellular uptake of the nanocarriers (Figure S4E). This functional evidence definitively confirms that the internalization of LNP‐OTP is actively driven by specific ligand‐receptor interactions rather than non‐specific endocytosis.

To demonstrate bone‐targeting capability in vivo, Cy5.5‐labeled LNPs were intravenously injected into mice bearing K7M2 orthotopic tibial tumors. Mice treated with mBAP1/siENO1@LNP‐OTP showed much stronger fluorescence signals at the tumor site; at 12 h post‐injection, the intensity was more than three times that of the mBAP1/siENO1@LNP group, indicating superior targeting ability (Figure 3J,K).

In vivo biodistribution was evaluated in the orthotopic K7M2‐luciferase tibial osteosarcoma model. After intravenous administration, mBAP1/siENO1@LNP‐OTP showed progressive fluorescence enrichment at the tibial tumor site, indicating preferential tumor accumulation of the OTP‐modified LNPs (Figure S5A,B). Moderate fluorescence signals were also observed in the lungs at early time points, which may be attributable to transient pulmonary retention after systemic nanoparticle administration and/or potential targeting of early pulmonary metastatic lesions in this highly lung‐metastatic K7M2 model. In addition, weak residual kidney signals were observed, likely reflecting partial renal clearance of fluorescent components. Therefore, the biodistribution data support preferential tumor accumulation of mBAP1/siENO1@LNP‐OTP, while also indicating expected systemic distribution and clearance patterns following intravenous administration.

Following the confirmation of targeted LNP internalization, we evaluated the co‐delivery efficiency of both therapeutic nucleic acids, a prerequisite for synergistic efficacy. LNPs were engineered to co‐encapsulate mCherry ‐labeled mBAP1 and Cy5‐labeled siENO1. Flow cytometry analysis revealed that approximately 84.4% of K7M2 cells were double‐positive for both fluorophores (Figure 3L,M). Notably, this dual‐internalization rate was comparable to the uptake efficiencies observed in the single‐cargo delivery controls.

Long‐term toxicity assessments revealed no significant changes in hematological parameters (white blood cell subtypes, RBC, WBC, and platelet counts) (Figure S5C, F‐I). Biochemical analysis confirmed that liver (ALT, AST, TBIL), heart (CK, CK‐MB), and kidney markers remained within physiological limits, as did serum protein levels (Figure S5D,E). These findings confirm that mBAP1/siENO1@LNP‐OTP possesses excellent biosafety and avoids immune overactivation. In summary, the OTP‐modified LNPs encapsulating ENO1 siRNA and BAP1 mRNA demonstrate precise targeting and effective gene regulation.

### 2.4 mBAP1/siENO1@LNP‐OTP Induces Synergistic Ferroptosis Through Complementary BAP1/SLC7A11 and ENO1/GPX4 Pathways

To verify the ferroptosis‐inducing effects of our dual‐delivery system, we performed a series of experiments (Figure 4A). First, mBAP1/siENO1@LNP‐OTP effectively inhibited the secretion of EVs‐ENO1 by K7M2 cells, with an efficacy superior to that of mBAP1/siENO1@LNP (Figure 4B). Flow cytometry indicated that mBAP1/siENO1@LNP‐OTP significantly upregulated lipid peroxidation levels (Figure 4C,D). Simultaneously, the intracellular GSSG proportion rose significantly, demonstrating a potent ferroptosis‐inducing effect (Figure 4E, Figure S6A). ROS detection showed that tumor cells treated with mBAP1/siENO1@LNP‐OTP experienced higher oxidative stress (Figure S6B,C). Furthermore, mBAP1/siENO1@LNP‐OTP markedly increased tumor cell death, indicating strong anti‐tumor cytotoxicity (Figure 4F,G).

**FIGURE 4:**
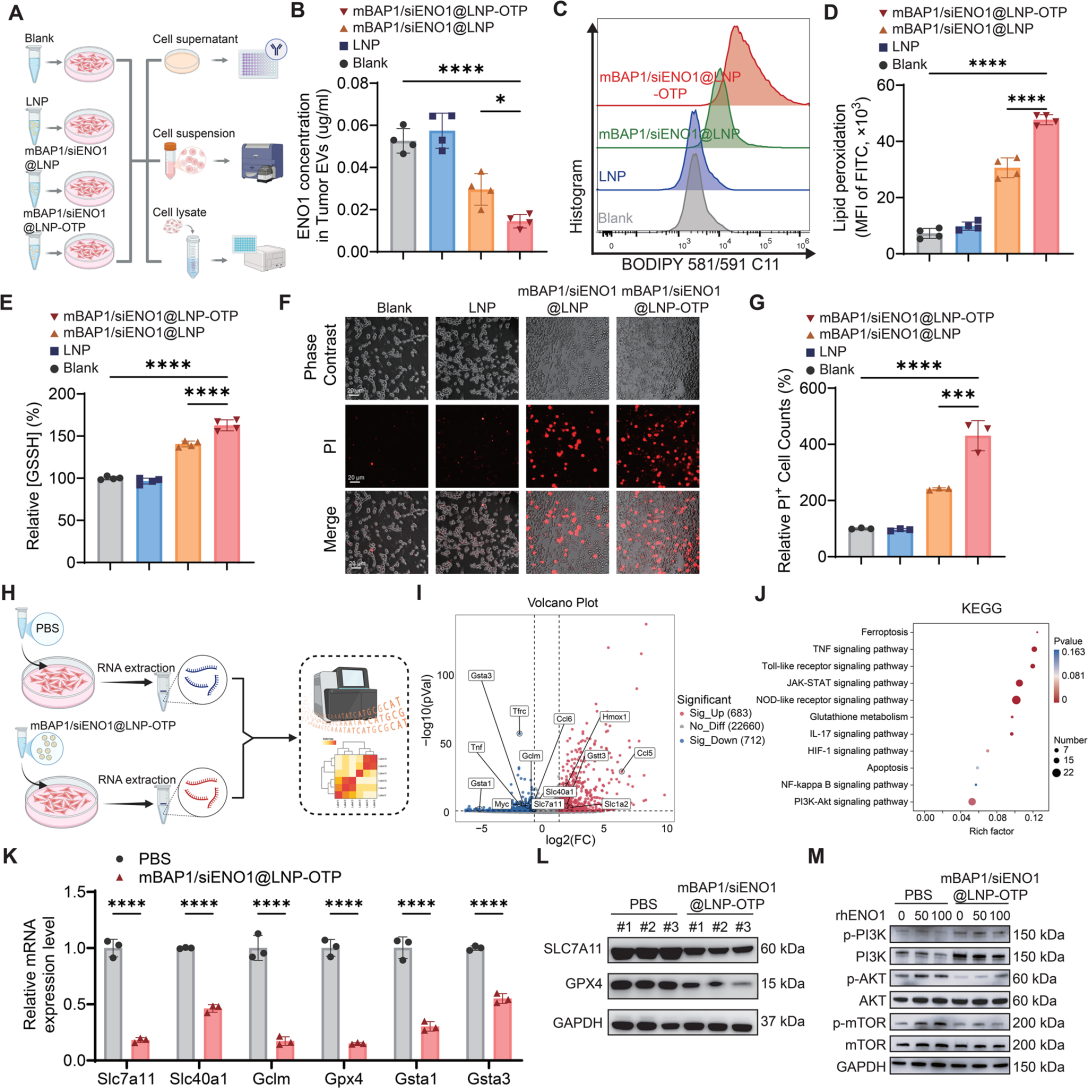
Targeted delivery of mBAP1/siENO1@LNP‐OTP inhibits EVs‐ENO1 and promotes ferroptosis in K7M2 cells. A: Experimental design. Schematic illustration of the experimental workflow for cell treatments (created with BioRender.com). B: Suppression of EVs‐ENO1. ELISA analysis of ENO1 levels in TEVs isolated from K7M2 cells following treatment with PBS, LNP, mBAP1/siENO1@LNP, or mBAP1/siENO1@LNP‐OTP for 24 h (n = 3). C–D: Lipid peroxidation analysis. Representative flow cytometry plots (C) and quantification (D) of lipid peroxidation changes in K7M2 cells stained with BODIPY 581/591 C11 following the indicated treatments (n = 3). E: Glutathione metabolism. Relative GSSG levels in K7M2 cells treated with PBS, LNP, mBAP1/siENO1@LNP, or mBAP1/siENO1@LNP‐OTP for 24 h (n = 3). F–G: Cell death analysis. Representative Propidium Iodide (PI) fluorescence staining images (F) and quantification of the percentage of PI^+^ cells (G) in K7M2 cells after 24 h of indicated treatments (n = 3). H: RNA‐seq workflow. Schematic workflow of RNA‐sequencing analysis in K7M2 cells after PBS or mBAP1/siENO1@LNP‐OTP treatment. (created with BioRender.com). I: Transcriptomic changes. Volcano plot showing differentially expressed genes (DEGs) in K7M2 cells treated with mBAP1/siENO1@LNP‐OTP compared to the PBS control group. J: Functional enrichment. KEGG pathway enrichment analysis of DEGs in K7M2 cells following mBAP1/siENO1@LNP‐OTP treatment. K: RT‐qPCR analysis of ferroptosis‐related gene mRNA expression in K7M2 cells treated with PBS or mBAP1/siENO1@LNP‐OTP (n = 3). L: Ferroptosis‐related protein expression. Western blot analysis of SLC7A11 and GPX4 levels in K7M2 cells following treatment with PBS or mBAP1/siENO1@LNP‐OTP. M: Signaling pathway analysis. Western blot analysis of key factors in the PI3K‐Akt signaling pathway in K7M2 cells treated with PBS or mBAP1/siENO1@LNP‐OTP. Data are presented as mean ± s.e.m. Statistical significance was determined by unpaired two‐tailed Student's t‐test for two‐group comparisons, one‐way ANOVA followed by Tukey's post hoc test for multiple‐group comparisons, and two‐way ANOVA for time‐course analyses. ns, not significant; *p < 0.05, **p < 0.01, ***p < 0.001, ****p < 0.0001.

To functionally validate ferroptosis as the predominant cell death modality, we performed pharmacological inhibitor rescue assays. As shown in Figure S6D, the reduction in cell viability induced by mBAP1/siENO1@LNP‐OTP was significantly rescued by the ferroptosis inhibitor Ferrostatin‐1 (Fer‐1), whereas the pan‐caspase apoptosis inhibitor Z‐VAD‐FMK failed to restore cell viability. Consistently, BODIPY 581/591 C11 staining showed that Fer‐1 co‐treatment markedly suppressed mBAP1/siENO1@LNP‐OTP‐induced intracellular lipid peroxidation (Figure S6E). In addition, the protective effects of K7M2‐derived EVs and ENO1‐CMVMs against cell death were largely abolished in the presence of Fer‐1, but remained evident after Z‐VAD‐FMK treatment (Figure S6F,G). Together, these functional rescue results provide strong evidence that the dual‐cargo delivery system predominantly induces ferroptotic cell death rather than apoptosis.

To explore the underlying mechanism, we performed high‐throughput transcriptome sequencing (RNA‐seq) on treated K7M2 cells (Figure 4H). Differential gene expression analysis revealed the downregulation of several ferroptosis‐regulating proteins, including Tfrc, Slc7a11, and Slc40a1 (Figure 4I, Figure S6H). Glutathione metabolism and transport molecules such as Gclm, Gsta1, and Gsta3 were also inhibited. KEGG enrichment analysis confirmed the activation of pathways related to Ferroptosis, Glutathione metabolism, and Apoptosis (Figure 4J, Figure S6I). Additionally, changes in the Nod‐like receptor, TNF, and NF‐kappa B signaling pathways suggested that tumor cell inflammation was altered by ferroptosis. qPCR validation confirmed that anti‐ferroptotic molecules like Tfrc, Slc7a11, and Gpx4 were significantly suppressed (Figure 4K). Although Tfrc mRNA expression was decreased after mBAP1/siENO1@LNP‐OTP treatment, FerroOrange staining showed that the intracellular labile Fe^2^^+^ level was not significantly altered (Figure S6J,K). These data suggest that the decrease in Tfrc expression did not lead to substantial depletion of the intracellular labile iron pool. Therefore, mBAP1/siENO1@LNP‐OTP‐induced ferroptosis is mainly attributable to impaired antioxidant defense through simultaneous suppression of the SLC7A11‐ and GPX4‐associated pathways, rather than increased iron uptake.

Western Blotting further showed that SLC7A11 and GPX4 protein expression was significantly inhibited by mBAP1/siENO1@LNP‐OTP, representing the collapse of tumor ferroptosis resistance (Figure 4L). Sequencing also suggested changes in the PI3K/Akt pathway, a key driver of tumor progression. We found that the phosphorylation levels of AKT and mTOR were significantly inhibited by mBAP1/siENO1@LNP‐OTP, even when supplemented with ENO1‐CMVMs (Figure 4M).

In summary, these findings indicate that this nanoplatform successfully dismantles the epigenetic and metabolic defense lines of tumor cells, inducing powerful anti‐tumor effects by reducing ferroptosis tolerance.

To further substantiate the mechanistic basis of the dual‐cargo strategy, we performed gain‐of‐function, loss‐of‐function, and rescue experiments to separately validate the BAP1/SLC7A11 and ENO1/GPX4 pathways. BAP1 overexpression in K7M2 cells markedly reduced SLC7A11 expression at both the mRNA and protein levels, supporting the ability of BAP1 restoration to suppress the SLC7A11‐associated antioxidant defense pathway (Figure S7A–C). In parallel, ENO1 knockdown significantly decreased GPX4 expression and enhanced lipid peroxidation, as indicated by increased BODIPY 581/591 C11 fluorescence and elevated 4‐HNE levels (Figure S7D–H). Conversely, ENO1 overexpression increased GPX4 expression and reduced lipid peroxidation (Figure S7I,M). Importantly, re‐expression of ENO1 in ENO1‐depleted cells restored GPX4 expression and reduced lipid ROS accumulation and 4‐HNE levels (Figure S7N–P). Together, these results support a dual‐pathway mechanism in which BAP1 restoration suppresses the SLC7A11‐associated antioxidant pathway, while ENO1 silencing impairs GPX4‐associated ferroptosis defense.

### 2.5 Targeted LNP Therapy Elicits Potent Anti‐Tumor Efficacy in Orthotopic Osteosarcoma Models

To evaluate the therapeutic potential of mBAP1/siENO1@LNP‐OTP, we established an orthotopic K7M2‐luci tibial tumor model in BALB/c mice (Figure 5A). Starting on day 5 post‐tumor implantation, mice were treated every three days via tail vein injection with PBS (G1), LNP (G2), mBAP1/siENO1@LNP (G3), or mBAP1/siENO1@LNP‐OTP (G4). Tumor growth curves showed that from day 12, the dual‐delivery system exhibited powerful efficacy, significantly inhibiting tumor growth (Figure 5B). Bioluminescence imaging confirmed that the G4 group had the weakest fluorescence, indicating the strongest tumor suppression compared to control and monotherapy groups (Figure 5C,D). On day 18, tumor weights were measured; G4 tumors were the smallest among the four groups, significantly smaller than G1, G2, and G3, demonstrating the superior therapeutic potential conferred by the OTP modification (Figure 5E,F).

**FIGURE 5:**
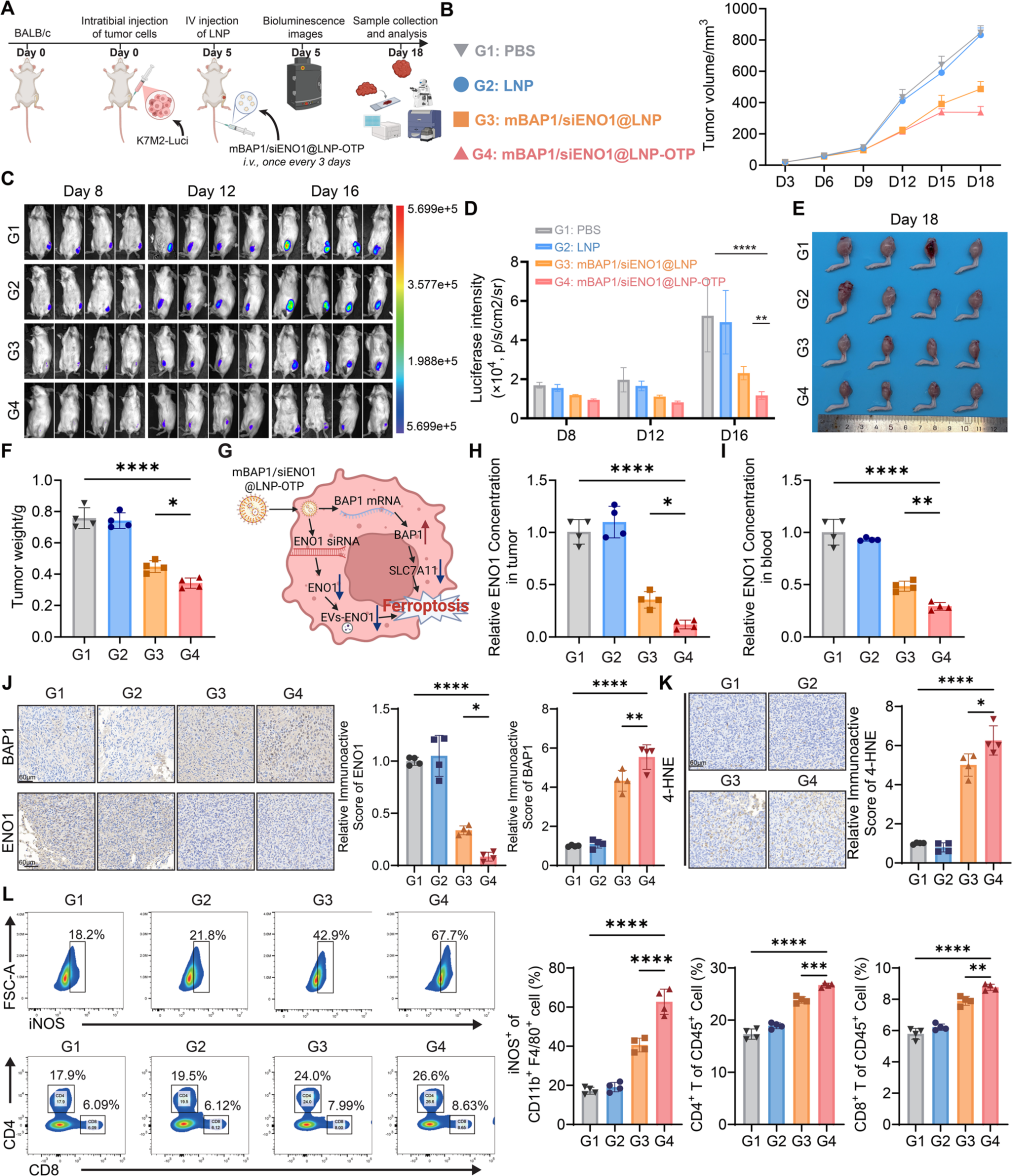
mBAP1/siENO1@LNP‐OTP suppresses primary osteosarcoma growth and modulates the tumor microenvironment in vivo. A: Therapeutic protocol. Schematic illustration of the intratibial osteosarcoma (OS) modeling and the subsequent mBAP1/siENO1@LNP‐OTP treatment regimen (created with BioRender.com). B: Tumor growth kinetics. In vivo tumor growth curves of K7M2 osteosarcoma in mice following treatment with PBS (G1), LNP (G2), mBAP1/siENO1@LNP (G3), or mBAP1/siENO1@LNP‐OTP (G4) (n = 4). C–D: Bioluminescence imaging. Representative in vivo bioluminescence images (C) and quantitative analysis of bioluminescence signal (D) in mice across the indicated treatment groups. Radiant efficiency is expressed as counts (n = 4). E–F: Tumor morphology and weight. Representative photographs of harvested intratibial tumors (E) and corresponding tumor weights (F) at the end of the study (n = 4). G: Schematic of sample collection. Schematic diagram illustrating the collection of tumor tissues and blood samples for downstream analysis. H: Intratumoral ENO1 levels. Relative ENO1 protein levels within the tumor tissues following the indicated treatments, as measured by ELISA (n = 4). I: Systemic ENO1 levels. Relative concentrations of circulating ENO1 in the blood of mice across treatment groups (n = 4). J: Immunohistochemical (IHC) analysis. Representative IHC staining images and corresponding quantitative analysis of BAP1 and ENO1 in tumor sections (n = 4). K: IHC analysis. Representative IHC staining images and corresponding quantitative analysis of 4‐HNE in tumor sections (n = 4). L: Immune cell analysis. Representative flow cytometry plots and statistical quantification of iNOS expression in CD11b^+^ F4/80^+^ macrophages and CD4/CD8 expression in CD3^+^CD45^+^ cells isolated from intratibial tumors (n = 4). Data are presented as mean ± s.e.m. Statistical significance was determined by unpaired two‐tailed Student's t‐test for two‐group comparisons, one‐way ANOVA followed by Tukey's post hoc test for multiple‐group comparisons, and two‐way ANOVA for time‐course analyses. ns, not significant; *p < 0.05, **p < 0.01, ***p < 0.001, ****p < 0.0001.

To further evaluate the individual contribution of each therapeutic cargo in vivo, we included single‐cargo control groups in the orthotopic K7M2‐luciferase osteosarcoma model. Both siENO1@LNP‐OTP and mBAP1@LNP‐OTP partially inhibited tumor progression compared with the PBS group, whereas the dual‐cargo formulation mBAP1/siENO1@LNP‐OTP produced a more pronounced anti‐tumor effect than either single‐cargo formulation. In vivo bioluminescence imaging showed that mBAP1/siENO1@LNP‐OTP resulted in the lowest tumor burden at Day 16, and tumor weight analysis at Day 18 further confirmed the enhanced therapeutic efficacy of the dual‐cargo formulation (Figure S8A–D). These results support the cooperative benefit of simultaneously restoring BAP1 and silencing ENO1 in the targeted LNP system.

Consistent with our design to inhibit EVs‐ENO1 secretion via ENO1 siRNA and weaken ferroptosis tolerance via BAP1 mRNA (Figure 5G), we found that G4 tumor tissues had significantly lower EVs‐ENO1 concentrations compared to all other groups (Figure 5H). Blood tests showed a similar drastic reduction in circulating EVs‐ENO1 (Figure 5I). Histological staining showed significantly decreased ENO1 expression and increased BAP1 positivity in G3 and G4 tumors (Figure 5J). Staining for 4‐HNE, a lipid peroxidation marker, revealed that G3 and G4 treatments induced significantly higher ferroptosis levels, resulting in tumor inhibition (Figure 5K). These results confirm that mBAP1/siENO1@LNP‐OTP successfully inhibits EVs‐ENO1 secretion and induces BAP1 expression to exert strong anti‐tumor effects.

Furthermore, we assessed immune activation within the TME. Flow cytometry showed that the proportions of M1 macrophages (iNOS^+^) and active T cells (CD4^+^, CD8^+^) were significantly upregulated in the G3 and G4 groups (Figure 5L). To further determine whether mBAP1/siENO1@LNP‐OTP treatment functionally activates anti‐tumor immunity, we performed additional cytokine/chemokine profiling and immune‐cell functional analyses using tumor tissues from PBS‐ and mBAP1/siENO1@LNP‐OTP‐treated mice. qPCR analysis showed that mBAP1/siENO1@LNP‐OTP treatment markedly upregulated the mRNA expression of several immune‐activating cytokines and chemokines, including Ccl2, Ccl5, Cxcl9, Cxcl10, Il‐12, Tnf‐α, and Ifn‐γ (Figure S8E,F). These molecules are associated with inflammatory macrophage activation, T‐cell recruitment, and effector immune responses, suggesting that the dual‐delivery system promotes a more immunostimulatory tumor microenvironment.

Consistently, flow cytometric analysis further demonstrated that mBAP1/siENO1@LNP‐OTP treatment increased the proportions of CD86^+^, iNOS^+^, and TNF‐α^+^ cells within the CD45^+^CD11b^+^F4/80^+^ macrophage population (Figure S8G–I), indicating enhanced pro‐inflammatory macrophage activation. In addition, mBAP1/siENO1@LNP‐OTP treatment increased the proportion of IFN‐γ^+^ CD8^+^ T cells (Figure S8J,K), further supporting enhanced effector T‐cell activation.

Together, these cytokine/chemokine and immune‐cell functional readouts demonstrate that mBAP1/siENO1@LNP‐OTP treatment not only induces ferroptotic tumor cell death but also activates anti‐tumor immune responses within the tumor microenvironment.

### 2.6 Synergistic Intervention Suppresses Pulmonary Metastasis and Reprograms the Immune Microenvironment

To evaluate the effect on advanced disease, we utilized a K7M2‐Luci tail vein injection‐induced lung metastasis model (Figure 6A). The bioluminescence intensity in the lungs of the G4 (mBAP1/siENO1@LNP‐OTP) group was significantly lower than that in the G1 (PBS), G2 (LNP), and G3 (mBAP1/siENO1@LNP) groups (Figure 6B,C). Upon harvesting lung tissues on day 16, we observed that G3 and G4 mice had smaller lung volumes and significantly fewer surface nodules (Figure 6D). H&E staining confirmed fewer metastatic nodules in the G3 and G4 groups, indicating a significant inhibition of metastasis (Figure 6E). Furthermore, the G4 group had the lowest EVs‐ENO1 concentration in lung tissue, significantly lower than the other three groups (Figure 6F). Blood tests showed identical trends (Figure 6G). Histological results showed inhibited ENO1 and induced BAP1 expression in G3 and G4 lung tissues (Figure 6J). 4‐HNE staining indicated higher ferroptosis activity in these groups. Flow cytometry analysis showed that the proportion of F4/80^+^iNOS^+^ (M1) macrophages in the mBAP1/siENO1@LNP‐OTP group was the highest, increasing by approximately 30% compared to the PBS group (Figure 6H,I).

**FIGURE 6:**
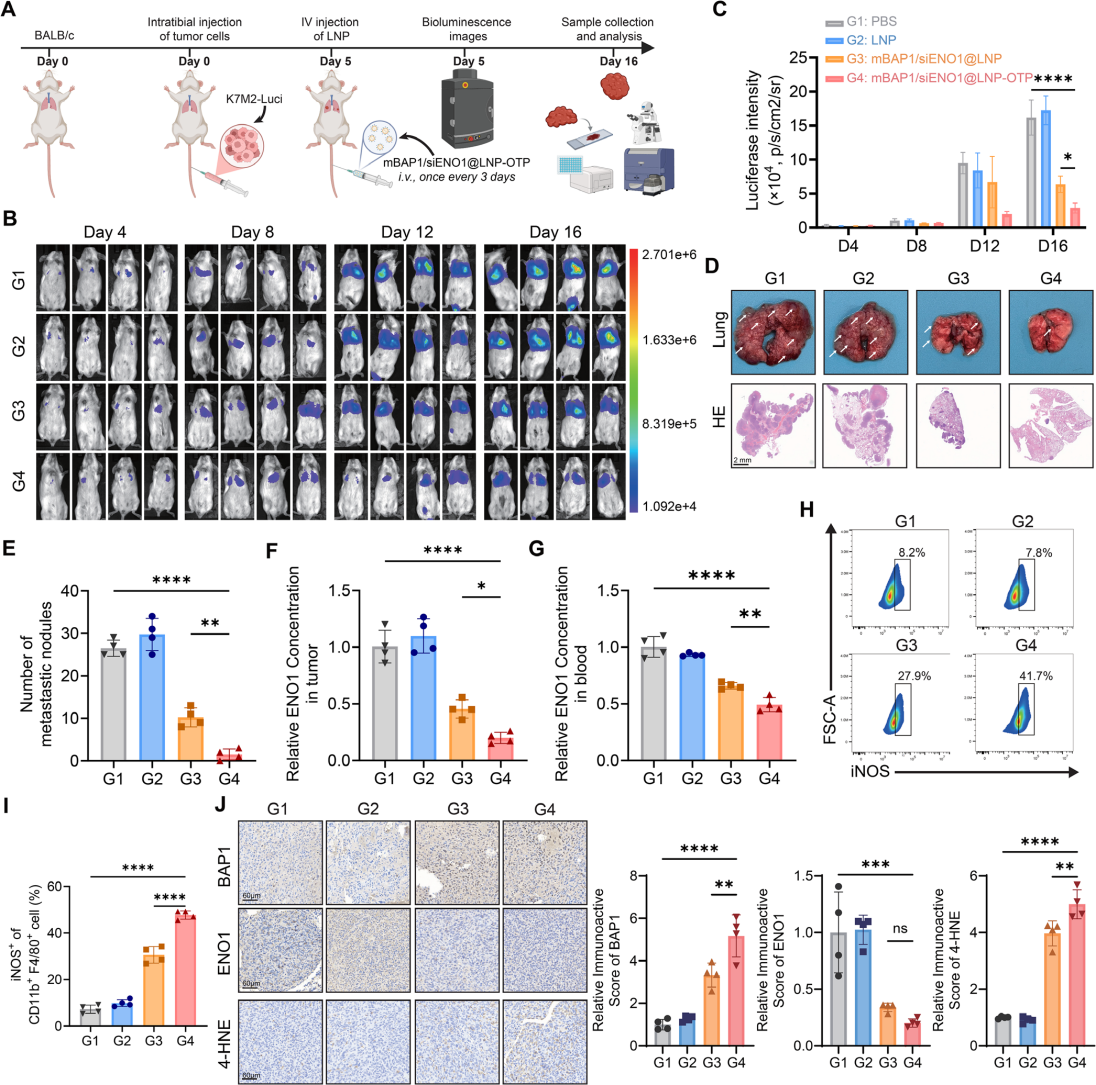
mBAP1/siENO1@LNP‐OTP inhibits lung metastasis and promotes an anti‐tumor immune microenvironment. A: Metastasis model protocol. Schematic illustration of the lung metastatic OS modeling and the timeline for mBAP1/siENO1@LNP‐OTP treatment (created with BioRender.com). B–C: Bioluminescence imaging of lung metastasis. Representative ex vivo bioluminescence images (B) and quantitative analysis of bioluminescence signal (C) in the lungs of mice across treatment groups. Color bar units are expressed as counts (n = 4). D–E: Evaluation of metastatic nodules. Representative photographs of harvested lungs and H&E‐stained lung sections (D) with corresponding quantification of metastatic nodules (E) per lung (n = 4). F: Intratumoral ENO1 expression. Relative ENO1 protein levels within the lung metastatic lesions following the indicated treatments (n = 4). G: Circulating ENO1 levels. Relative ENO1 concentrations in the blood of mice bearing lung metastases after treatment with the indicated formulations (n = 4). H–I: Macrophage polarization in lung lesions. Representative flow cytometry plots (H) and statistical analysis (I) of iNOS^+^ cells among CD45^+^CD11b^+^F4/80^+^ macrophages harvested from lung metastatic tumors (n = 4). J: IHC profiling of metastatic lesions. Representative IHC images and quantitative analysis of BAP1, ENO1, and 4‐HNE (lipid peroxidation marker) expression in lung metastatic tumor tissues across different groups (n = 4). Data are presented as mean ± s.e.m. Statistical significance was determined by unpaired two‐tailed Student's t‐test for two‐group comparisons, one‐way ANOVA followed by Tukey's post hoc test for multiple‐group comparisons, and two‐way ANOVA for time‐course analyses. ns, not significant; *p < 0.05, **p < 0.01, ***p < 0.001, ****p < 0.0001.

In summary, these findings demonstrate that mBAP1/siENO1@LNP‐OTP effectively targets OS cells to inhibit growth and metastasis both in vitro and in vivo. Simultaneously, it activates immune activity within the TME, amplifying anti‐tumor efficacy. Our dual‐delivery system shows great therapeutic potential and holds promise as a treatment strategy for solid tumors.

## 3 Discussion

This study provides a comprehensive analysis of how OS cells transmit ferroptosis resistance signals via EVs. Our experimental data clearly indicate that ENO1 is highly enriched in tumor‐derived EVs, a phenomenon that is further amplified under ferroptotic stress as an adaptive defense mechanism to protect the tumor population from oxidative damage. In terms of therapeutic strategy, we utilized a LNP platform to achieve the co‐delivery of the epigenetic regulator BAP1 and the metabolic oncogene ENO1. Our therapeutic platform, mBAP1/siENO1@LNP‐OTP, first achieves the potent inhibition of ENO1 expression alongside the robust upregulation of BAP1 in tumor cells. This dual‐action modulation leads to a substantial depletion of ENO1 within TEVs, effectively dismantling the tumor's intrinsic ferroptosis resistance system and markedly sensitizing the cells to ferroptotic cell death.

In recent years, mounting evidence has demonstrated that EVs play a crucial role in regulating cellular ferroptosis [50]. Tao L et al. discovered that skeletal muscle cells release FNDC5/irisin‐enriched exosomes during exercise to inhibit osteoblast ferroptosis, thereby exerting a bone‐protective effect [51]. A recent study on acute myeloid leukemia (AML) indicated that AML cell‐derived exosomes suppress lipid peroxidation and ferroptosis in the cells themselves, aiding their escape from chemotherapy and immunotherapy [52]. Furthermore, in an epithelial ovarian cancer study, Jiang Y et al. also found ACSL4‐enriched EVs aid ovarian cancer cells in evading ferroptosis therapy [30]. Similarly, in this study, we found that ferroptosis induction leads to reactive release of increased extracellular vesicles by tumor cells, enhancing their ferroptosis tolerance. Subsequently, inhibiting tumor cell secretion of EVs effectively enhanced the killing efficacy of ferroptosis inducers. Using prior mass spectrometry sequencing data of osteosarcoma EVs, we identified ENO1 as enriched in TEVs. Indeed, in a recent clinical study, researchers detected ENO1 in extracellular vesicles from serum samples of Ewing sarcoma patients, with its levels significantly decreasing post‐treatment, reflecting the diagnostic and therapeutic value of ENO1^+^ extracellular vesicles [53]. Our findings demonstrate that administration of ENO1‐CMVMs significantly enhances ferroptosis resistance and promotes the proliferation and metastasis of osteosarcoma cells. In vivo administration of ENO1‐CMVMs effectively attenuated the tumor‐suppressive effects of FINs, resulting in an increased tumor burden in murine models. Furthermore, ENO1‐CMVM treatment promoted an immunosuppressive TME characterized by increased M2‐like macrophage polarization and reduced infiltration of activated T cells. The role of vesicle‐associated ENO1 in enhancing ferroptosis resistance significantly expands our understanding of the non‐canonical functions of this classical metabolic enzyme and provides new theoretical support for targeting ENO1 in solid tumor therapy.

Importantly, the selection of BAP1 as a therapeutic mRNA cargo is based on its unique position as an upstream epigenetic regulator of ferroptosis susceptibility. Unlike conventional ferroptosis inducers that act on downstream metabolic enzymes such as GPX4 or directly modulate lipid peroxidation, these approaches often suffer from transient efficacy and limited tumor specificity. In contrast, BAP1 functions at the transcriptional level by modulating chromatin accessibility and repressing SLC7A11 expression, thereby enabling sustained reprogramming of cellular redox homeostasis. Moreover, BAP1 is a well‐characterized tumor suppressor frequently downregulated in multiple malignancies, including osteosarcoma, where its loss contributes to aggressive tumor behavior and metabolic reprogramming. Therefore, BAP1 restoration not only sensitizes tumor cells to ferroptosis but also simultaneously reactivates intrinsic tumor suppressive pathways, providing a dual therapeutic advantage that cannot be achieved by conventional ferroptosis‐targeting agents. This upstream and pleiotropic regulatory nature of BAP1 makes it a superior therapeutic candidate for mRNA‐based delivery systems aimed at achieving durable ferroptosis sensitization in solid tumors.

As a nascent yet potent anti‐tumor strategy, ferroptosis‐based therapy has emerged as a focal point of intensive investigation within the global scientific community [54, 55]. However, the effective clinical translation of ferroptosis‐based therapies faces several pivotal challenges. These include inter‐ and intra‐tumor heterogeneity, the requirement for enhanced drug specificity, and a notable paucity of validated biomarkers, all of which are particularly pronounced in the context of solid tumors [56]. The selection of osteosarcoma in this study is also relevant to the current challenge of heterogeneous ferroptosis sensitivity among different tumors. OS is characterized by high metabolic activity, dysregulated iron homeostasis, and elevated oxidative stress, rendering it relatively susceptible to ferroptosis induction [57, 58, 59]. Nevertheless, accumulating evidence indicates that OS cells can acquire adaptive ferroptosis resistance through multiple antioxidant and metabolic defense mechanisms, highlighting the complexity and heterogeneity of ferroptosis sensitivity [60, 61]. Our findings regarding EV‐mediated ferroptosis resistance may therefore provide mechanistic insights that are applicable to other ferroptosis‐responsive malignancies.

Ferroptosis therapy is a double‐edged sword: while inducing ferroptotic cell death in malignant tumors, it also risks damaging normal tissues, highlighting the critical importance of targeting [15, 62]. Based on our prior research, the high affinity of targeting peptides for tumor cells ensures precise localization of nucleic acid payloads to primary lesions and metastatic sites within complex systemic circulation [47, 63]. This targeted approach minimizes non‐specific accumulation in organs of the mononuclear phagocyte system (MPS), such as the liver and spleen [64]. The superior tissue penetration and cellular internalization efficiency represent the core advantages of this platform over conventional systemic chemotherapy and non‐targeted gene therapies [65, 66, 67]. Recent studies highlight that intrinsic and acquired resistance to ferroptosis in tumors is a major obstacle to the development of ferroptosis therapies [68, 69]. Increasing evidence indicates that tumors commonly evolve active mechanisms to tolerate ferroptosis, primarily through enhanced antioxidant system compensation and protective effects of the TME [18]. Addressing this challenge, our study uniquely identified tumor cells secreting ENO1 to upregulate ferroptosis resistance, offering novel insights into overcoming tumor ferroptosis tolerance. Furthermore, most current FINs face pharmacokinetic issues hindering delivery, such as poor water solubility, low in vivo stability, and insufficient tumor accumulation. This study developed an LNP platform targeting OS for tumor suppressor gene delivery. The observed decrease in Tfrc mRNA may represent an adaptive feedback response of tumor cells attempting to restrict additional iron uptake under ferroptotic stress. However, intracellular Fe^2^^+^ measurement showed that the labile iron pool was maintained at a basal level after treatment. Thus, the therapeutic effect of mBAP1/siENO1@LNP‐OTP does not rely on increasing intracellular iron availability. Instead, simultaneous impairment of the SLC7A11‐ and GPX4‐associated antioxidant defenses, through BAP1 restoration and ENO1 silencing, respectively, renders tumor cells unable to efficiently detoxify lipid peroxides, allowing the existing intracellular iron pool to support ferroptosis execution. By integrating peptides with strong OS binding affinity onto the LNP surface through PEGylated lipids, these therapeutic agents were directly incorporated into the lipid matrix [70]. This strategy successfully delivered ferroptosis‐regulating genes precisely to primary tumor sites and metastases, avoiding toxicity risks in normal tissues while achieving highly efficient tumor killing.

Despite these promising findings, several limitations warrant consideration. First, while the K7M2 syngeneic model recapitulates key features of aggressive OS, validation in PDX or spontaneous metastasis models would further strengthen translational relevance. Second, although OTP enhances tumor targeting, receptor identification and binding specificity require deeper investigation to optimize delivery efficiency. Third, long‐term safety and potential immune responses to repeated LNP administration need further evaluation before clinical translation. Fourth, ENO1‐CMVMs used in the mechanistic and functional validation experiments represent a reductionist artificial vesicle system rather than native tumor‐derived EVs. Although this approach enables controlled evaluation of ENO1 delivered in a membrane‐vesicle context while reducing interference from other endogenous EV cargos, CMVMs do not fully reproduce the biogenesis, membrane composition, cargo heterogeneity, surface protein organization, or biological behavior of naturally secreted TEVs. Therefore, the ENO1‐CMVM experiments should be interpreted as complementary mechanistic evidence for the function of vesicle‐associated ENO1 rather than as a complete recapitulation of native TEV biology.

## 4 Conclusion

This study identifies EVs‐ENO1 as a critical mediator of ferroptosis resistance in OS and reveals a previously unrecognized mechanism by which tumor cells exploit intercellular communication to evade oxidative cell death and remodel the tumor microenvironment. By simultaneously restoring BAP1 and silencing ENO1, our OS‐targeted mBAP1/siENO1@LNP‐OTP nanoplatform coordinately modulates the BAP1/SLC7A11 and ENO1/GPX4 pathways, triggers robust lipid peroxidation‐dependent cell death, and reprograms anti‐tumor immunity. This dual epigenetic–metabolic strategy not only suppresses primary tumor growth but also markedly inhibits pulmonary metastasis with minimal systemic toxicity. Collectively, our findings establish ferroptosis modulation as a viable therapeutic avenue in OS and provide a precision nanomedicine framework that may be broadly applicable to other ferroptosis‐resistant malignancies.

## 5 Methods

### 5.1 Experimental Section

#### 5.1.1 Cell Culture

The BALB/c mouse osteosarcoma‐derived K7M2 cells were kindly provided by Prof. Zhaoming Ye (School of Medicine, Zhejiang University, China). Cells were routinely cultured in DMEM with 10% fetal bovine serum (FBS) and penicillin/streptomycin in a humidified 37°C incubator with 5% CO_2_. Cells were routinely tested negative for mycoplasma contamination. For EV isolation from culture supernatants, cells were changed to medium with EV‐depleted FBS as previously reported [71].

#### 5.1.2 Cell Migration and Invasion Assays

A wound healing assay was used to measure the migration of differently treated K7M2 cells. After the cell proliferation in the 6‐well plate reached approximately 90% confluence, a 100 µL pipette tip was used to evenly scrape the cell layer to form a scratch. After that, scraped cell debris was gently removed with PBS. Then, serum‐free medium was added, and pictures of the scratch were immediately photographed under a microscope. Every 12 h, pictures of the cell scratches were taken again, and the wound area was analyzed using ImageJ software.

In addition, Transwell chambers with 8‐µm pore‐size membranes (Corning Life Sciences, USA) were used to evaluate the migration or invasion of cancer cells. For invasion assays, the membranes were coated with Matrigel. Briefly, 600 µL of medium containing 10% FBS was added to the lower chamber. A total of 1×10^5^ differently treated K7M2 cells were resuspended in 200 µL of serum‐free medium and seeded into the upper chamber. After incubation for 24–36 h, the cells were washed with PBS, fixed with 4% paraformaldehyde for 20 min, and stained with 0.1% crystal violet for 5–10 min. Cells remaining on the upper surface of the membrane were gently removed with a cotton swab, whereas cells that migrated or invaded through the membrane were imaged and quantified using an inverted microscope.

#### 5.1.3 Detection of Lipid Peroxidation

Lipid peroxidation levels were visualized using the BODIPY 581/591 C11 probe (Beyotime, China). Briefly, a 2 µM working solution was prepared by diluting the 2 mM stock solution 1,000‐fold in PBS. K7M2 cells seeded in 6‐well plates were washed once with PBS and then incubated with 1 mL of the staining solution at 37°C for 10–30 min in a humidified atmosphere. Following incubation, the cells were rinsed twice with PBS to remove excess probe. Subsequently, 2 mL of PBS was added to each well, and the intracellular fluorescence shifts were captured via confocal laser scanning microscopy (Leica TCS SP8).

#### 5.1.4 Intracellular GSH and GSSG Assay

The intracellular levels of total glutathione (T‐GSH) and oxidized glutathione (GSSG) were determined using a GSH/GSSG Assay Kit (Beyotime, S0053) following the manufacturer's instructions. Briefly, freshly collected K7M2 cells were resuspended in three volumes of protein removal reagent M, subjected to two rapid freeze‐thaw cycles using liquid nitrogen and a 37°C water bath, and centrifuged at 10,000 × g for 10 min at 4°C to obtain the supernatant. To measure GSSG specifically, a portion of the sample was treated with GSH scavenger and auxiliary reagents at 25°C for 60 min to eliminate endogenous GSH.

Subsequently, the samples and standards were incubated with the T‐GSH detection working solution for 5 min, followed by the addition of NADPH (0.5 mg/mL). The absorbance at 412 nm was recorded using a microplate reader. T‐GSH and GSSG concentrations were calculated based on the respective standard curves. The reduced GSH content was determined using the formula: GSH = Total Glutathione – (GSSG × 2). All results were normalized to the total protein concentration of the samples.

#### 5.1.5 Pharmacological Inhibition and Rescue Assays

K7M2 cells were seeded in 96‐well plates and treated with mBAP1/siENO1@LNP‐OTP in the presence or absence of Fer‐1 (1 µM) or Z‐VAD‐FMK (20 µM). After treatment, cell viability was measured using the CCK‐8 assay according to the manufacturer's instructions. For lipid peroxidation analysis, cells were incubated with BODIPY 581/591 C11 probe, washed with PBS, and analyzed by fluorescence microscopy or flow cytometry. The ability of Fer‐1 or Z‐VAD‐FMK to reverse mBAP1/siENO1@LNP‐OTP‐induced cytotoxicity and lipid ROS accumulation was used to distinguish ferroptosis from apoptosis.

#### 5.1.6 Plasmids, Cloning and Lentivirus Production

To generate stable knockdown cell lines, shRNAs against murine ENO1 were inserted into the pLVX‐shRNA2‐LucPuro vector. The shRNAs used were as follows: ENO1 shRNA: CCGGGCTTCTGTTCGACCTGACAAACTCGAGTTTGTCAGGTCG AACAGAAGCTTTTTG. Lentivirus packing murine ENO1‐expression vectors (pCDH‐EF1‐Puro) were obtained from Synbio Tech. (Suzhou, China). Lentivirus was produced in 293T cells. Cells were infected with viral particles and selected by the indicated concentration of puromycin for 7 days. The expression vectors containing ENO1 or Flag‐tagged ENO1 were purchased from Synbio Tech. (Suzhou, China). 293T cells were transfected with Lipofectamine 3000 according to the manufacturer's instructions. The siRNA sequences are listed in the Supplementary Table S2.

#### 5.1.7 Western Blot (WB) Analysis

Briefly, cells or EVs were lysed in RIPA buffer (CWBIO, China) containing protease inhibitor and phosphatase inhibitor (Selleck, China). Protein was separated by SDS–PAGE and then transferred to PVDF membranes (Millipore). After blocking with 5% nonfat milk, the membranes were probed with the indicated primary antibody overnight at 4°C and then incubated with the corresponding secondary antibodies. Bands were detected by Chemidoc Touch (Bio–Rad) with Clarity Western ECL substrate (Bio–Rad). The antibodies are listed in the Supplementary Table S3.

#### 5.1.8 Isolation and Characterization of EVs

EVs from cell lines were isolated by ultracentrifugation as described previously [71]. Briefly, the cell culture supernatant was collected and centrifuged at 2000×g for 10 min and then at 10,000×g for 30 min. The supernatant was passed through a 0.22 µm filter and then ultracentrifuged at 100,000×g for 2 h. The pellet was resuspended in PBS and re‐ultracentrifuged at 100,000×g for 2 h. The EV pellet was resuspended in PBS and used immediately or stored at ‐80°C.

The isolated TEVs were characterized by TEM, NTA, Western blotting, and zeta potential analysis. For TEM analysis, TEV suspensions were loaded onto copper grids, negatively stained with uranyl acetate, and observed under a transmission electron microscope to assess vesicle morphology. Particle size distribution and particle concentration were measured using NTA. The expression of canonical EV markers, including CD9 and TSG101, and the negative marker Calnexin was examined by Western blotting to verify EV identity and assess cellular contamination. The zeta potential of isolated TEVs was measured using a dynamic light scattering analyzer. Total protein concentration of TEVs was determined using a BCA protein assay kit (CWBIO, China), and TEVs were used at the indicated concentrations in subsequent experiments.

#### 5.1.9 EV Treatment In Vitro

EVs were isolated by differential ultracentrifugation. The concentration of EVs was determined using a BCA protein assay. For functional experiments, recipient tumor cells were treated with TEVs at a final concentration of 10 µg/mL EV protein unless otherwise indicated.

#### 5.1.10 ELISA

The concentrations of target proteins were determined using commercially available ELISA kits according to the manufacturer's protocols. Briefly, 100 µL of either standards or samples were loaded into pre‐coated microplates and incubated for 2 h at room temperature. Following three washes to remove unbound substances, the plates were incubated with a specific biotin‐labeled detection antibody. Subsequently, Streptavidin‐HRP was introduced for 30 min. After a final wash cycle, TMB substrate was added for color development, and the reaction was halted using a stop solution. The absorbance was measured at 450 nm using a microplate reader. Target concentrations were then interpolated from a standard curve generated for each assay.

#### 5.1.11 Animal Studies

All in vivo experiments were approved by the Animal Ethics Committee of the Second Affiliated Hospital, Zhejiang University School of Medicine Female BALB/c mice (4 weeks old) were obtained from Zhejiang University Medical Laboratory Animal Center.

An in vivo orthotopic OS model was established by intratibial injection of K7M2 cells. Briefly, 1 × 10^6^ K7M2 cells suspended in 10 µL PBS were injected into the proximal tibia.

Tumor‐bearing mice were monitored until the experimental endpoints specified for each study, as described below.

For the ENO1‐CMVM orthotopic gain‐of‐function experiment, mice received ENO1‐CMVMs intravenously every three days at a dose of 50 µg per mouse. Where indicated, FIN was administered intraperitoneally at 5 mg/kg. Mice were euthanized on day 16 after tumor inoculation, and the primary tumors were harvested for subsequent tumor assessment, histological analysis, immunohistochemistry, and flow cytometric analysis.

For the ENO1‐CMVM lung colonization experiment, 1 × 10^6^ K7M2 cells were injected via the tail vein. Mice received ENO1‐CMVMs intravenously every three days at a dose of 50 µg per mouse. Where indicated, FIN was administered intraperitoneally at 5 mg/kg. On day 14, the mice were euthanized and the lungs were harvested immediately. Fresh portions of lung tissue were processed into single‐cell suspensions on the day of collection for flow cytometric analysis. The remaining lung tissues were fixed in 4% paraformaldehyde for 24 h and subsequently subjected to paraffin embedding, sectioning, H&E staining, and immunohistochemical analysis.

#### 5.1.12 Bioluminescence Imaging

In vivo bioluminescence imaging (BLI) was performed using the AniView system (Biolight, China). Mice were anesthetized with 2% isoflurane and subsequently received an intraperitoneal injection of D‐luciferin (15 mg/mL, 100 µL per mouse). To evaluate the primary tumor burden and metastatic progression, the total flux (photons/second) within a standardized region of interest (ROI) was quantified using the integrated AniView analysis software.

#### 5.1.13 Immunohistochemistry (IHC) and Immunofluorescence (IF)

For IF staining, cells were plated in 24‐well plates, washed with phosphate‐buffered saline (PBS), fixed in 4% formaldehyde for 15 min at room temperature, and then permeabilized in 0.05% Triton X‐100 in PBS for 10 min, followed by 10% BSA blocking for 20 min at room temperature. Cells were incubated overnight at 4°C in blocking buffer containing antibody. Cells were washed with PBS and incubated with Alexa Fluor 555‐labeled donkey anti‐rabbit IgG for 2 h at room temperature. Cells were washed with PBS, and high‐resolution images captured using microscopy (DMi8, Leica).

For immunohistochemical staining, formalin‐fixed paraffin‐embedded human osteosarcoma tissues or mouse tumor tissues were cut into 5‐µm thick serial sections and subsequently incubated with antibodies overnight at 4°C. The slides were then incubated with HRP‐conjugated secondary antibodies, visualized using DAB, and counterstained with hematoxylin. Images were acquired using a microscope (Leica) and Leica Application Suite X v3.7.4 software.

Immunohistochemistry analysis was evaluated in five randomly selected fields of vision. Semi‐quantitative histoscore (H‐score) was calculated by quantification of the percentage of positively stained cells using ImageJ (version 1.8.0) software. Staining intensity was scored from 1 to 3. The following formula was used to calculate final H‐score as follows: H‐score = (low %) × 1 + (medium %) × 2 + (high %) × 3. Arg1, F4/ 80, CD86, iNOS, or CD206 positive cells were counted based on the positive staining in each microscopic field. The optimal cut‐off values of the H‐scores from human osteosarcoma specimens for Kaplan–Meier analyses were determined by X‐Tile software.

#### 5.1.14 Isolation of Tumor Tissue‐Derived EVs

Tumor tissue‐derived EVs were isolated according to established protocols with minor modifications. Briefly, fresh tumor tissues were washed three times with ice‐cold PBS to remove blood contamination and then minced into small fragments (approximately 1–2 mm^3^).

The tissue fragments were incubated with collagenase type IV (1 mg/mL) and DNase I (0.1 mg/mL) in serum‐free DMEM at 37°C for 30 min with gentle agitation. The resulting cell suspension and interstitial fluid were filtered through a 70‐µm cell strainer to remove large debris.

The filtrate was then subjected to a series of differential centrifugation steps: 2,000×g for 10 min, followed by 10,000×g for 30 min at 4°C to eliminate dead cells and larger microvesicles. The supernatant was passed through a 0.22‐µm filter and subsequently ultracentrifuged at 100,000×g for 2 h (Optima L‐100 XP, Beckman Coulter). The pellet was resuspended in PBS, followed by a second round of ultracentrifugation at 100,000×g for 2 h for purification. Finally, the EV‐enriched pellet was resuspended in PBS and either used immediately or stored at ‐80°C. The protein concentration of the isolated EVs was determined using a BCA assay (CWBIO, China).

#### 5.1.15 Flow Cytometry Analysis of Infiltrated Immune Cells

Tumor tissues were lavaged with normal saline and then cut into small pieces. Tissues were homogenized using a gentleMACS dissociator (Miltenyi Biotec) in solution with 2 mg/mL collagenase type I (Sigma), 2 mg/mL collagenase type IV (Sigma), and 1 mg/mL DNase (Sigma). The suspension was filtered through a 70‐µm cell strainer. For analysis of spleen immune cells, the spleen was smashed through a 70‐µm nylon mesh in 1640 medium. Cell suspensions were lysed in RBC lysis buffer (Biolegend) to remove RBCs and then washed, counted, and resuspended in PBS. Suspensions were first blocked with Fc‐block (CD16/CD32) and then incubated with the indicated primary antibodies for 30 min at room temperature. After washing with PBS twice, the cells were analyzed by a CytoFLEX (Beckman Coulter). Gating was determined by the fluorescence minus one approach. Data were analyzed with CytExpert V2.4 or FlowJo V10 software. The antibodies used for flow cytometry are listed in the Key Resources Table.

#### 5.1.16 Preparation and Characterization of ENO1‐Loaded Cell Membrane‐Derived Vesicle Mimetics

ENO1‐loaded cell membrane‐derived vesicle mimetics (ENO1‐CMVMs) were prepared using an extrusion‐based method. Briefly, K7M2 cells were collected and disrupted to isolate cell membrane fractions. The isolated membrane fractions were washed with PBS and co‐extruded with rhENO1 through polycarbonate membranes to generate ENO1‐CMVMs. To remove unencapsulated free rhENO1, the post‐extrusion mixture was purified by repeated ultrafiltration using 100 kDa MWCO centrifugal filters and washed with PBS for three cycles. The encapsulation efficiency of rhENO1 was determined by measuring the amount of input and unencapsulated rhENO1 using a BCA protein assay. Particle size distribution and zeta potential were measured by dynamic light scattering, and vesicle morphology was examined by transmission electron microscopy. For in vitro dose‐response experiments, ENO1‐CMVM concentrations were normalized to the encapsulated rhENO1 content after removal of unencapsulated protein and are therefore reported as rhENO1‐equivalent concentrations (µg/mL). This normalization was used only for the in vitro dose‐response experiments; in vivo dosing was reported directly as the administered ENO1‐CMVM dose (µg per mouse).

#### 5.1.17 OS‐Targeting Peptide Synthesis and Purification

The OS‐targeting peptide was synthesized via standard Fmoc‐based solid‐phase peptide synthesis (SPPS) on Fmoc‐Lys(Dde)‐Wang resin (0.3 mmol/g). Each coupling cycle employed 4.0 equivalents of Fmoc‐protected amino acids, activated by TBTU and HOBt in DMF at 35°C for 40 min. Fmoc deprotection was achieved using 20% piperidine in DMF (v/v). Following the assembly of the backbone (QYWNIQNGPYGGKKKK), the Dde protecting group on the C‐terminal lysine was selectively removed with 2% hydrazine hydrate in DMF. Palmitic acid was then conjugated to the ε‐amino group via TBTU/HOBt activation. Subsequently, azidoacetic acid was coupled to the N‐terminus to introduce the azide moiety for subsequent click chemistry. The reaction progress was monitored by the Kaiser test. The final peptide‐resin was cleaved using a TFA‐based cocktail (82.5% TFA, 5% H_2_O, 5% thioanisole, 5% phenol, 2.5% EDT) for 3 h, precipitated in ice‐cold diethyl ether, and purified via RP‐HPLC (Kromasil C18 column). The identity and purity of the peptide were confirmed by analytical HPLC and ESI‐MS.

#### 5.1.18 Preparation of mBAP1/siENO1@LNP‐OTP

The targeting ligand, DSPE‐PEG2K‐Pep, was synthesized via strain‐promoted azide‐alkyne cycloaddition (SPAAC). DSPE‐PEG2K‐DBCO (50 nmol) and N‐terminal azidoacetylated peptide (50 nmol) were reacted in a DMSO/EtOH/water co‐solvent system at 37°C for 1 h under constant agitation (1100 rpm). The resulting conjugate was purified by dialysis (MWCO: 3.5 kDa) and validated by UV‐vis spectrophotometry (observing the depletion of the DBCO absorbance at 309 nm), MALDI‐TOF MS, and ^1^H NMR.

Targeted LNPs were formulated using a microfluidic platform (NanoAssemblr Ignite). For dual‐cargo encapsulation, mBAP1 mRNA and siENO1 were mixed at a mass ratio of 2:1 in 50 mM citrate buffer (pH 4.0) before microfluidic mixing. The organic phase, containing the ionizable lipid DLin‐MC3‐DMA, cholesterol, DSPC, DMG‐PEG2K, and DSPE‐PEG2K‐Pep, was rapidly mixed with the aqueous nucleic acid phase at a flow rate ratio (FRR) of 3:1 and a total flow rate (TFR) of 12 mL/min. The LNPs were prepared at an N/P ratio of 6:1, where N represents the protonatable amine groups of DLin‐MC3‐DMA and P represents the phosphate groups of the nucleic acid cargos.

The optimized lipid molar composition of mBAP1/siENO1@LNP‐OTP was DLin‐MC3‐DMA: DSPC: cholesterol: DMG‐PEG2K: DSPE‐PEG2K‐Pep = 50: 10: 38.5: 1.0: 0.5 (mol%), with a total PEG‐lipid content of 1.5 mol%. For non‐targeted LNPs (mBAP1/siENO1@LNP), DSPE‐PEG2K‐Pep was omitted and replaced with DMG‐PEG2K, while maintaining the same total PEG‐lipid content (DLin‐MC3‐DMA: DSPC: cholesterol: DMG‐PEG2K = 50: 10: 38.5: 1.5 mol%).

The incorporation of DSPE‐PEG2K‐Pep at 0.5 mol% was designed to provide sufficient surface exposure of the OTP ligand while minimizing potential steric hindrance caused by excessive PEGylation. The combination of DMG‐PEG2K and DSPE‐PEG2K‐Pep enabled a balance between nanoparticle stability and active tumor targeting, as DMG‐PEG2K provides transient PEG shielding whereas DSPE‐PEG2K‐Pep ensures stable presentation of the targeting peptide.

Following formulation, the LNP suspension was dialyzed against PBS (pH 7.4) for 24 h to remove ethanol and adjust the pH to physiological levels. Finally, the LNPs were concentrated via centrifugal filtration (100 kDa MWCO) and characterized for size, zeta potential, and encapsulation efficiency (EE%) using the RiboGreen assay.

#### 5.1.19 Characterization

The morphological characteristics of the nanovesicles were visualized via transmission electron microscopy (TEM; JEM‐1200EX, Japan). To evaluate the hydrodynamic diameter and surface charge (zeta potential), dynamic light scattering (DLS) and electrophoretic mobility analyses were performed using a Zetasizer Nano ZS (Malvern Panalytical, UK). Furthermore, the optical properties and UV–vis absorption spectra of the various nanovesicle formulations were characterized using a SHIMADZU UV‐3600 Plus spectrophotometer (Japan).

#### 5.1.20 siRNA and mRNA Stability

To assess the protective capacity of different nanocarriers, siRNA and mRNA‐loaded complexes were incubated in PBS (pH 7.4) supplemented with 50% FBS at 37°C. At designated time points (0, 3, 6, 9, 12, 18, and 24 h), the nanostructures were disrupted with 1% Triton X‐100 to trigger the release of encapsulated nucleic acids. The structural integrity of the liberated siRNA and mRNA was subsequently resolved via 2% agarose gel electrophoresis to visualize potential degradation.

#### 5.1.21 Cell Cytotoxicity Evaluation

The biocompatibility of various formulations was scrutinized using the CCK‐8 assay. K7M2 cells were seeded in 96‐well plates at a density of 5×10^3^ cells/well and cultured overnight for attachment. Subsequently, the medium was replaced with fresh formulations (PBS, LNP, mBAP1/siENO1@LNP, and mBAP1/siENO1@LNP‐OTP) at concentrations ranging from 2 to 12 µg/mL. After 24 h of exposure, cell viability was quantified by measuring the absorbance of the CCK‐8 reagent according to the manufacturer's instructions.

#### 5.1.22 Cellular Uptake of Nanovesicles

For cellular uptake studies, K7M2 cells were plated in confocal dishes (2.0×10^5^ cells/dish) and grown to 50–70% confluence. Cells were treated with DiO‐labeled mBAP1/siENO1@LNP‐OTP (1 µg/mL) for 30 min at 37°C. After removing unbound vesicles through triple PBS rinsing, the cells were fixed and nuclei were counterstained with DAPI. Fluorescent distribution was visualized using a Leica TCS SP8 confocal laser scanning microscope.

#### 5.1.23 In Vivo Fluorescence Imaging of mBAP1/siENO1@LNP‐OTP

To evaluate the targeting capability in vivo, an orthotopic bone tumor model was established by microinjecting K7M2‐luci cells (1×10^6^ in 10 µL PBS) into the right tibial marrow cavity. Ten days later, mice received a tail vein injection of Cy5.5‐labeled mBAP1/siENO1@LNP or mBAP1/siENO1@LNP‐OTP (100 µg). The systemic distribution of the nanovesicles was monitored at 12, 24, and 48 h post‐injection using an IVIS system under isoflurane anesthesia. For ex vivo analysis, the primary organs and tumor tissues were excised at intervals up to 72 h to quantify the localized fluorescence intensity.

#### 5.1.24 In Vivo Therapeutic Efficacy

The therapeutic efficacy of the LNP formulations was evaluated in both orthotopic and experimental lung metastasis K7M2‐luciferase osteosarcoma models.

For the orthotopic osteosarcoma model, K7M2‐luci cells (1 × 10^6 cells in 10 µL PBS) were injected into the tibial marrow cavity. Five days after tumor inoculation, mice were randomly allocated into four groups (n = 4): PBS, LNP, mBAP1/siENO1@LNP, and mBAP1/siENO1@LNP‐OTP. The respective formulations (100 µg per dose) were administered intravenously every three days. Tumor progression was monitored longitudinally by measuring tumor volumes every two days and by bioluminescence imaging at the indicated time points. On day 18, the mice were euthanized and the primary tumors were harvested for tumor‐weight measurement and subsequent molecular, histological, and immune analyses.

For the experimental lung metastasis model, 1 × 10^6 K7M2‐luci cells were injected via the tail vein. Mice were allocated to the same four treatment groups (PBS, LNP, mBAP1/siENO1@LNP, and mBAP1/siENO1@LNP‐OTP) and received the corresponding formulations (100 µg per dose) intravenously every three days. On day 16, the mice were euthanized and the lungs were harvested for ex vivo bioluminescence imaging, gross metastatic nodule assessment, histological and immunohistochemical analyses, EVs‐ENO1 measurement, and flow cytometric analysis of tumor‐infiltrating immune cells.

#### 5.1.25 Quantitative Real‐Time PCR

Total RNA was extracted using RNAiso reagent (Takara). Reverse transcription was achieved using a cDNA Synthesis Kit (Takara). mRNA levels were measured using gene‐specific primers and SYBR Premix Ex TaqTM kits (Takara). Data were acquired using Real‐Time PCR software (version 2.4). Expression results were normalized to β‐actin levels. Primers were from PrimerBank or bought from OriGene and can be found in Supplementary Table S1.

#### 5.1.26 RNA Sequencing (RNA‐Seq)

Transcriptome sequencing was conducted by Sinotech Genomics Co., Ltd. (Shenzhen, China) on the Illumina NovaSeq 600 platform following standard protocols. Total cellular RNA was isolated using TRIzol reagent (Invitrogen), and its purity, concentration, and integrity were rigorously validated to meet the requirements for high‐quality library construction. Each sample yielded approximately 6 GB of clean data. Sequences were aligned to the mouse reference genome (GRCm38/mm10), and gene expression levels were quantified and normalized as Fragments Per Kilobase of transcript per Million mapped reads (FPKM).

#### 5.1.27 GEO and TARGET Data Analysis

The results of this study were partly generated by analysis of data from TARGET and GEO datasets: GSE237033, GSE21257, GSE33382, and GSE14359. GSEA was performed using GSEA software [72]. CIBERSORT was used to estimate abundances of different cells in a mixed immune cell population [73, 74].

#### 5.1.28 Statistical Analysis

No data transformation or normalization was performed for routine in vitro and in vivo experiments unless otherwise specified. No data points were excluded from the analyses, and no statistical methods were used to predetermine sample size. Data are presented as mean ± standard error of mean (SEM). The sample size (n) for each experiment is indicated in the corresponding figure legends and represents the number of biologically independent samples or animals.

Statistical analyses were performed using GraphPad Prism 10.0 (GraphPad Software, San Diego, CA, USA). Comparisons between two groups were performed using unpaired two‐tailed Student's t‐test. Comparisons among three or more groups were performed using one‐way analysis of variance (ANOVA) followed by Tukey's multiple comparisons test. For experiments involving two independent variables, two‐way ANOVA followed by Tukey's multiple comparisons test was used where appropriate. Statistical significance was defined as p < 0.05 (α = 0.05). Exact *p* values or significance levels are provided in the corresponding figures and figure legends. The assumptions of normal distribution and homogeneity of variance were considered based on the experimental design and the sample sizes commonly used in the field.

## Author Contributions

**Yechao Shen**: conceptualization, investigation, methodology, validation, visualization, writing – original draft, formal analysis, supervision, project administration, data curation. **Zhenguo Cao**: conceptualization, methodology, investigation, data curation. **Zenan Wang**: data curation, supervision, formal analysis, validation. **Liang Chen**: validation, methodology, formal analysis. **Jiahao Zhang**: validation, formal analysis, investigation. **Linjie Zeng**: investigation, validation, formal analysis. **Boren Tan**: validation, formal analysis. **Hengyuan Li**: validation, formal analysis. **Jiashan Lai**: validation, formal analysis. **Jiangchu Lei**: validation, formal analysis. **Fanglu Chen**: validation, formal analysis. **Peng Lin**: supervision, investigation, writing – review and editing. **Zhenxuan Shao**: writing – review and editing, conceptualization, investigation, funding acquisition, supervision. **Zhan Wang**: funding acquisition, writing – review and editing, supervision.

## Funding

This work was supported by National Natural Science Foundation of China (Nos. 82103499, 82503698, 82373164), Zhejiang Provincial Natural Science Foundation (LQ22H160040), Medical Science and Technology Project of Zhejiang Province (2024KY1025), the Postdoctoral Fellowship Program of CPSF under Grant Number GZB20240660 and China Postdoctoral Science Foundation (No. 2024M762845).

## Conflicts of Interest

The authors declare no conflicts of interest.

## Ethics Statement

All animal experiments were approved by Animal Ethics Committee, the Second Affiliated Hospital of Zhejiang University School of Medicine (AIRB‐2021‐1122). The Zhejiang University Animal Ethics Committee reviewed animal welfare during the application process of animal experiments and animal experiments. All patients recruited for this study followed a protocol approved by Human Research Ethics Committee, the Second Affiliated Hospital of Zhejiang University School of Medicine (IRB‐2021‐812) and obtained informed written consent.

## Supporting information




**Supporting File 1**: advs78242‐sup‐0001‐SuppMat.docx.

**Supporting File 2**: advs78242‐sup‐0002‐SuppMat.pdf.

## Data Availability

The data that support the findings of this study are available from the corresponding author upon reasonable request.
