# Supplementary material for: A Dual‐Delivery System Designed to Overcome EV‐Mediated Ferroptosis Resistance for Efficient Solid Tumor Therapy
Source: Adv Sci (Weinh). 2026 Oct 11:e78242. Online ahead of print. doi: 10.1002/advs.78242 (PMC13655257; doi:10.1002/advs.78242)
Supplement: Supplementary file 1 [file ADVS-9999-e78242-s002.pdf]

```
Reference sequence (1): Human_ENO1
Identities normalised by aligned length.
Colored by: identity

                     cov     pid   1 [         .         .         .         .         :         .         .         . 80
1 Human_ENO1      100.0% 100.0%      MSILKIHAREIFDSRGNPTVEVDLFTSKGLFRAAVPSGASTGIYEALELRDNDKTRYMGKGVSKAVEHINKTIAPALVSK
2 Mouse_ENO1       99.5%  94.7%      -SILRIHAREIFDSRGNPTVEVDLYTAKGLFRAAVPSGASTGIYEALELRDNDKTRFMGKGVSQAVEHINKTIAPALVSK
  consensus/100%                     .SIL+IHAREIFDSRGNPTVEVDLaTuKGLFRAAVPSGASTGIYEALELRDNDKTRaMGKGVSpAVEHINKTIAPALVSK
  consensus/90%                      .SIL+IHAREIFDSRGNPTVEVDLaTuKGLFRAAVPSGASTGIYEALELRDNDKTRaMGKGVSpAVEHINKTIAPALVSK
  consensus/80%                      .SIL+IHAREIFDSRGNPTVEVDLaTuKGLFRAAVPSGASTGIYEALELRDNDKTRaMGKGVSpAVEHINKTIAPALVSK
  consensus/70%                      .SIL+IHAREIFDSRGNPTVEVDLaTuKGLFRAAVPSGASTGIYEALELRDNDKTRaMGKGVSpAVEHINKTIAPALVSK

                     cov     pid  81         .         1         .         .         .         .         :         . 160
1 Human_ENO1      100.0% 100.0%      KLNVTEQEKIDKLMIEMDGTENKSKFGANAILGVSLAVCKAGAVEKGVPLYRHIADLAGNSEVILPVPAFNVINGGSHAG
2 Mouse_ENO1       99.5%  94.7%      KVNVVEQEKIDKLMIEMDGTENKSKFGANAILGVSLAVCKAGAVEKGVPLYRHIADLAGNPEVILPVPAFNVINGGSHAG
  consensus/100%                     KlNVsEQEKIDKLMIEMDGTENKSKFGANAILGVSLAVCKAGAVEKGVPLYRHIADLAGNsEVILPVPAFNVINGGSHAG
  consensus/90%                      KlNVsEQEKIDKLMIEMDGTENKSKFGANAILGVSLAVCKAGAVEKGVPLYRHIADLAGNsEVILPVPAFNVINGGSHAG
  consensus/80%                      KlNVsEQEKIDKLMIEMDGTENKSKFGANAILGVSLAVCKAGAVEKGVPLYRHIADLAGNsEVILPVPAFNVINGGSHAG
  consensus/70%                      KlNVsEQEKIDKLMIEMDGTENKSKFGANAILGVSLAVCKAGAVEKGVPLYRHIADLAGNsEVILPVPAFNVINGGSHAG

                     cov     pid 161         .         .         .         2         .         .         .         . 240
1 Human_ENO1      100.0% 100.0%      NKLAMQEFMILPVGAANFREAMRIGAEVYHNLKNVIKEKYGKDATNVGDEGGFAPNILENKEGLELLKTAIGKAGYTDKV
2 Mouse_ENO1       99.5%  94.7%      NKLAMQEFMILPVGASSFREAMRIGAEVYHNLKNVIKEKYGKDATNVGDEGGFAPNILENKEALELLKTAIAKAGYTDQV
  consensus/100%                     NKLAMQEFMILPVGAusFREAMRIGAEVYHNLKNVIKEKYGKDATNVGDEGGFAPNILENKEuLELLKTAIuKAGYTDpV
  consensus/90%                      NKLAMQEFMILPVGAusFREAMRIGAEVYHNLKNVIKEKYGKDATNVGDEGGFAPNILENKEuLELLKTAIuKAGYTDpV
  consensus/80%                      NKLAMQEFMILPVGAusFREAMRIGAEVYHNLKNVIKEKYGKDATNVGDEGGFAPNILENKEuLELLKTAIuKAGYTDpV
  consensus/70%                      NKLAMQEFMILPVGAusFREAMRIGAEVYHNLKNVIKEKYGKDATNVGDEGGFAPNILENKEuLELLKTAIuKAGYTDpV

                     cov     pid 241         :         .         .         .         .         3         .         . 320
1 Human_ENO1      100.0% 100.0%      VIGMDVAASEFFRSGKYDLDFKSPDDPSRYISPDQLADLYKSFIKDYPVVSIEDPFDQDDWGAWQKFTASAGIQVVGDDL
2 Mouse_ENO1       99.5%  94.7%      VIGMDVAASEFYRSGKYDLDFKSPDDPSRYITPDQLADLYKSFVQNYPVVSIEDPFDQDDWGAWQKFTASAGIQVVGDDL
  consensus/100%                     VIGMDVAASEFaRSGKYDLDFKSPDDPSRYIoPDQLADLYKSFlpsYPVVSIEDPFDQDDWGAWQKFTASAGIQVVGDDL
  consensus/90%                      VIGMDVAASEFaRSGKYDLDFKSPDDPSRYIoPDQLADLYKSFlpsYPVVSIEDPFDQDDWGAWQKFTASAGIQVVGDDL
  consensus/80%                      VIGMDVAASEFaRSGKYDLDFKSPDDPSRYIoPDQLADLYKSFlpsYPVVSIEDPFDQDDWGAWQKFTASAGIQVVGDDL
  consensus/70%                      VIGMDVAASEFaRSGKYDLDFKSPDDPSRYIoPDQLADLYKSFlpsYPVVSIEDPFDQDDWGAWQKFTASAGIQVVGDDL

                     cov     pid 321         .         .         :         .         .         .         .         4 400
1 Human_ENO1      100.0% 100.0%      TVTNPKRIAKAVNEKSCNCLLLKVNQIGSVTESLQACKLAQANGWGVMVSHRSGETEDTFIADLVVGLCTGQIKTGAPCR
2 Mouse_ENO1       99.5%  94.7%      TVTNPKRIAKAASEKSCNCLLLKVNQIGSVTESLQACKLAQSNGWGVMVSHRSGETEDTFIADLVVGLCTGQIKTGAPCR
  consensus/100%                     TVTNPKRIAKAssEKSCNCLLLKVNQIGSVTESLQACKLAQuNGWGVMVSHRSGETEDTFIADLVVGLCTGQIKTGAPCR
  consensus/90%                      TVTNPKRIAKAssEKSCNCLLLKVNQIGSVTESLQACKLAQuNGWGVMVSHRSGETEDTFIADLVVGLCTGQIKTGAPCR
  consensus/80%                      TVTNPKRIAKAssEKSCNCLLLKVNQIGSVTESLQACKLAQuNGWGVMVSHRSGETEDTFIADLVVGLCTGQIKTGAPCR
  consensus/70%                      TVTNPKRIAKAssEKSCNCLLLKVNQIGSVTESLQACKLAQuNGWGVMVSHRSGETEDTFIADLVVGLCTGQIKTGAPCR

                     cov     pid 401         .         .         .   ] 434
1 Human_ENO1      100.0% 100.0%      SERLAKYNQLLRIEEELGSKAKFAGRNFRNPLAK
2 Mouse_ENO1       99.5%  94.7%      SERLAKYNQILRIEEELGSKAKFAGRSFRNPLA-
  consensus/100%                     SERLAKYNQlLRIEEELGSKAKFAGRsFRNPLA.
  consensus/90%                      SERLAKYNQlLRIEEELGSKAKFAGRsFRNPLA.
  consensus/80%                      SERLAKYNQlLRIEEELGSKAKFAGRsFRNPLA.
  consensus/70%                      SERLAKYNQlLRIEEELGSKAKFAGRsFRNPLA.
```

MView 1.67, Copyright © 1997-2020 Nigel P. Brown
