# Supplementary material for: A Dual‐Delivery System Designed to Overcome EV‐Mediated Ferroptosis Resistance for Efficient Solid Tumor Therapy
Source: Adv Sci (Weinh). 2026 Oct 11:e78242. Online ahead of print. doi: 10.1002/advs.78242 (PMC13655257; doi:10.1002/advs.78242)
Supplement: Supplementary file 2 [file ADVS-9999-e78242-s001.docx]

**Supplement Materials**

**Supplement Figures**

 **Figure S1. Characterization of K7M2-derived EVs and ferroptosis-related responses across tumor cells and immune cells.**

A: RSL3-induced cytotoxicity. Cell viability of K7M2 cells treated with indicated concentrations of the ferroptosis inducer RSL3 (n=3).

B: Representative transmission electron microscopy (TEM) image showing the vesicular morphology of K7M2-derived EVs. Scale bar, 200 nm.

C: Nanoparticle tracking analysis (NTA) showing the particle size distribution and concentration of K7M2-derived EVs.

D: Western blot analysis of EV-positive markers TSG101 and CD9 and the negative marker Calnexin in K7M2 cells and K7M2-derived EVs.

E: Zeta potential analysis of K7M2-derived EVs.

F-G: EV secretion under ferroptosis stress. Relative concentration of EVs secreted by Lewis Lung Carcinoma (LLC) cells (F) and 4T1 breast cancer cells (G) following treatment with varying concentrations of RSL3 (n=3).

H-I: Lipid peroxidation ratios. Relative ratio of oxidized to reduced lipid states in K7M2 cells (H) and macrophages (I) treated with media containing or lacking TEVs (n=3).

J-K: GSH levels in K7M2 and Macrophages. Relative levels of reduced glutathione (GSH) in K7M2 cells (J) and macrophages (K) across the indicated treatment groups (n=3).

L-M: Lipid peroxidation in Neutrophils. Representative flow cytometry plots (L) and statistical analysis (M) of lipid peroxidation changes in neutrophils using BODIPY™ 581/591 C11 staining (n=3).

N–O: Glutathione status in Neutrophils. Relative levels of oxidized glutathione disulfide (GSSG) (N) and reduced glutathione (GSH) (O) in neutrophils across different treatment groups (n=3).

Data are presented as mean ± s.e.m. Statistical significance was determined by unpaired two-tailed Student’s t-test for two-group comparisons, one-way ANOVA followed by Tukey’s post hoc test for multiple-group comparisons, and two-way ANOVA for time-course analyses. ns, not significant; *P < 0.05, **P < 0.01, ***P < 0.001, ****P < 0.0001.

**Figure S2. Bioinformatic analysis of ENO1 and its secretion in different cancer models.**

A: Gene Ontology (GO) enrichment analysis of differentially enriched proteins identified from the osteosarcoma-derived EV proteomic dataset. The enriched GO terms include biological processes associated with metabolic regulation, cellular stress responses, and redox-related pathways.

B: Kyoto Encyclopedia of Genes and Genomes (KEGG) pathway enrichment analysis of differentially enriched proteins from the osteosarcoma-derived EV proteomic dataset, showing enrichment of pathways related to tumor metabolism, stress adaptation, and ferroptosis-associated biological processes.

C: Pan-cancer analysis of ENO1. Expression profiling of ENO1 across various cancer types based on the TCGA dataset.

D: ENO1 expression in Osteosarcoma (OS). Differential expression analysis of ENO1 between OS tumor tissues and normal tissues using the TARGET-OS dataset.

E-F: ENO1 and OS Metastasis. Comparative analysis of ENO1 expression in tumor tissues from patients with metastatic vs. primary OS based on the TARGET-OS (E) and GSE237033 (F) datasets.

G: Representative immunohistochemical staining images and quantification of ENO1 expression in tumor tissues from metastatic and non-metastatic osteosarcoma patients in a 184-patient hospital cohort.

H: Prognostic value of ENO1. Kaplan-Meier survival analysis showing the correlation between ENO1 expression and prognosis in OS patients (TARGET-OS dataset).

I-J: Secreted EV-ENO1 levels. Concentration of ENO1 within EVs derived from LLC lung cancer cells (I) and 4T1 breast cancer cells (J) treated with indicated concentrations of RSL3.

Data are presented as mean ± s.e.m. Statistical significance was determined by unpaired two-tailed Student’s t-test for two-group comparisons, one-way ANOVA followed by Tukey’s post hoc test for multiple-group comparisons, and two-way ANOVA for time-course analyses. ns, not significant; *P < 0.05, **P < 0.01, ***P < 0.001, ****P < 0.0001.

**Figure S3. Flow cytometry gating strategies and characterization of ENO1-CMVMs.**

A: Macrophage gating strategy. Representative flow cytometry gating hierarchy used for the identification and analysis of macrophage populations.

B: T cell gating strategy. Representative flow cytometry gating hierarchy used for the identification and analysis of T cell subsets.

C: Verification of Flag-tagged ENO1. Western blot analysis confirming the transfection efficiency and expression of the ENO1-Flag construct.

D: Representative TEM image showing the vesicle-like morphology of ENO1-CMVMs.

E: Particle size distribution of ENO1-CMVMs measured by dynamic light scattering. F: Zeta potential of ENO1-CMVMs.

G: Encapsulation efficiency (EE) of rhENO1 in ENO1-CMVMs after removal of free rhENO1.

H: Macrophage polarization in primary tumors. Representative flow cytometry plots and statistical analysis of iNOS expression in CD11b^+^ F4/80^+^ macrophages harvested from K7M2 intratibial tumors on day 16 (n = 5).

I: Macrophage polarization in metastatic tumors. Representative flow cytometry plots and statistical analysis of iNOS expression in CD11b^+^ F4/80^+^ macrophages harvested from K7M2 lung metastatic lesions on day 14 (n = 5).

Data are presented as mean ± s.e.m. Statistical significance was determined by unpaired two-tailed Student’s t-test for two-group comparisons, one-way ANOVA followed by Tukey’s post hoc test for multiple-group comparisons, and two-way ANOVA for time-course analyses. ns, not significant; *P < 0.05, **P < 0.01, ***P < 0.001, ****P < 0.0001.

**Figure S4. Additional characterization and OTP-mediated uptake validation of mBAP1/siENO1@LNP-OTP.**

A: Characterization of LNP formulations. Summary of the physical properties (size, polydispersity index, and zeta potential) of the various Lipid Nanoparticle (LNP) formulations.

B: Mass spectrometry of OTP. Mass spectrometry (MS) report confirming the identity and purity of the OTP targeting ligand.

C: In vitro cytotoxicity. Cell viability of K7M2 cells treated with indicated concentrations of mBAP1/siENO1@LNP (n = 3).

D: Cellular uptake quantification. Quantitative analysis of the fluorescence intensity of LNP, mBAP1/siENO1@LNP, and mBAP1/siENO1@LNP-OTP internalized by K7M2 cells (n = 5).

E: Representative confocal microscopy images and quantification of DiO fluorescence intensity in K7M2 cells after incubation with DiO-labeled LNP-OTP, with or without pretreatment with excess free OTP peptide. Scale bar, 100 μm.

Data are presented as mean ± s.e.m. Statistical significance was determined by unpaired two-tailed Student’s t-test for two-group comparisons, one-way ANOVA followed by Tukey’s post hoc test for multiple-group comparisons, and two-way ANOVA for time-course analyses. ns, not significant; *P < 0.05, **P < 0.01, ***P < 0.001, ****P < 0.0001.

**Figure S5. Biodistribution and biosafety evaluation of mBAP1/siENO1@LNP-OTP.**

A: In vivo biodistribution. Representative ex vivo fluorescence images showing the distribution of mBAP1/siENO1@LNP-OTP in major organs at indicated time points post-injection.

B: Organ-specific accumulation. Quantitative analysis of fluorescence intensity in major organs and tumor sites to evaluate the targeting efficiency of mBAP1/siENO1@LNP-OTP (n = 3).

C: Hematological analysis (Differential counts). Percentages of neutrophils (NEU%), lymphocytes (LYM%), monocytes (MON%), eosinophils (EOS%), and basophils (BAS%) in mice following treatment with PBS or mBAP1/siENO1@LNP-OTP (n = 3).

D: Serum biochemistry (Liver and Heart). Serum levels of alanine aminotransferase (ALT), aspartate aminotransferase (AST), alkaline phosphatase (ALP), creatine kinase (CK), and creatine kinase-myocardial band (CK-MB) in treated mice (n = 3).

E: Serum biochemistry (Kidney and Metabolism). Serum levels of total bilirubin (TBIL), creatinine (CR), and uric acid (UA) evaluating renal and metabolic safety (n = 3).

F-I: Complete blood count (CBC) and protein. Analysis of red blood cells (RBC), white blood cells (WBC), platelets (PLT), and hemoglobin (HGB) levels in mice treated with PBS or mBAP1/siENO1@LNP-OTP (n = 3).

Data are presented as mean ± s.e.m. Statistical significance was determined by unpaired two-tailed Student’s t-test for two-group comparisons, one-way ANOVA followed by Tukey’s post hoc test for multiple-group comparisons, and two-way ANOVA for time-course analyses. ns, not significant; *P < 0.05, **P < 0.01, ***P < 0.001, ****P < 0.0001.

**Figure S6. Ferroptosis rescue validation, oxidative stress profiling, and intracellular Fe²⁺ analysis.**

A: Intracellular antioxidant status. Relative levels of GSH in K7M2 cells across the indicated treatment groups.

B–C: Reactive oxygen species (ROS) analysis. Representative flow cytometry plots (B) and statistical quantification (C) of total ROS levels in K7M2 cells using fluorescent probes across different treatment groups (n = 3).

D: Cell viability of K7M2 cells treated with PBS or mBAP1/siENO1@LNP-OTP in the presence or absence of Ferrostatin-1 (Fer-1) or Z-VAD-FMK, as determined by CCK-8 assay.

E: Representative BODIPY 581/591 C11 staining images showing intracellular lipid peroxidation in K7M2 cells treated with PBS or mBAP1/siENO1@LNP-OTP under control, Fer-1, or Z-VAD-FMK conditions.

F: Cell viability of K7M2 cells treated with PBS or K7M2-derived EVs in the presence or absence of Fer-1 or Z-VAD-FMK.

G: Cell viability of K7M2 cells treated with PBS or ENO1-CMVMs (0.2 μg/mL rhENO1 equivalent) in the presence or absence of Fer-1 or Z-VAD-FMK.

H: Differentially expressed gene (DEG) heatmap. Heatmap showing the expression profiles of DEGs in K7M2 cells following treatment with mBAP1/siENO1@LNP-OTP as determined by RNA-sequencing.

I: Gene Ontology (GO) enrichment. GO analysis highlighting the biological processes, molecular functions, and cellular components enriched in the DEGs of K7M2 cells after mBAP1/siENO1@LNP-OTP treatment.

J: Representative flow cytometry histograms of FerroOrange fluorescence in control and mBAP1/siENO1@LNP-OTP-treated K7M2 cells (J).

K: Quantification of intracellular Fe²⁺ levels based on the mean fluorescence intensity (MFI) of FerroOrange.

Data are presented as mean ± s.e.m. Statistical significance was determined by unpaired two-tailed Student’s t-test for two-group comparisons, one-way ANOVA followed by Tukey’s post hoc test for multiple-group comparisons, and two-way ANOVA for time-course analyses. ns, not significant; *P < 0.05, **P < 0.01, ***P < 0.001, ****P < 0.0001.

**Figure S7. Mechanistic validation of the BAP1/SLC7A11 and ENO1/GPX4 ferroptosis-regulatory pathways.**

A) qPCR analysis of BAP1 mRNA expression in K7M2 cells after BAP1 overexpression.

B) qPCR analysis of SLC7A11 mRNA expression in K7M2 cells after BAP1 overexpression.

C) Western blot analysis of BAP1 and SLC7A11 protein expression in control and BAP1-overexpressing K7M2 cells. GAPDH was used as the loading control.

D) qPCR analysis confirming ENO1 knockdown efficiency in K7M2 cells.

E) qPCR analysis of GPX4 mRNA expression after ENO1 knockdown.

F) Western blot analysis of ENO1 and GPX4 protein expression in control and ENO1-knockdown K7M2 cells. GAPDH was used as the loading control.

G) Quantification of 4-HNE levels in the culture supernatant after ENO1 knockdown.

H) Representative BODIPY 581/591 C11 staining images showing lipid peroxidation in control and ENO1-knockdown K7M2 cells. Green fluorescence indicates oxidized BODIPY C11, and red fluorescence indicates reduced BODIPY C11.

I) qPCR analysis confirming ENO1 overexpression efficiency in K7M2 cells.

J) qPCR analysis of GPX4 mRNA expression after ENO1 overexpression.

K) Western blot analysis of ENO1 and GPX4 protein expression in control and ENO1-overexpressing K7M2 cells. GAPDH was used as the loading control.

L) Quantification of 4-HNE levels in the culture supernatant after ENO1 overexpression.

M) Representative BODIPY 581/591 C11 staining images showing lipid peroxidation in control and ENO1-overexpressing K7M2 cells.

N) Western blot analysis of ENO1 and GPX4 protein expression in control, LNP-treated, and ENO1-rescue K7M2 cells. GAPDH was used as the loading control.

O) Flow cytometry analysis and quantification of lipid peroxidation using BODIPY 581/591 C11 staining in control, LNP-treated, and ENO1-rescue K7M2 cells.

P) Quantification of 4-HNE levels in the culture supernatant of control, LNP-treated, and ENO1-rescue K7M2 cells.

Data are presented as mean ± s.e.m. Statistical significance was determined by unpaired two-tailed Student’s t-test for two-group comparisons, one-way ANOVA followed by Tukey’s post hoc test for multiple-group comparisons, and two-way ANOVA for time-course analyses. ns, not significant; *P < 0.05, **P < 0.01, ***P < 0.001, ****P < 0.0001.

**Figure S8. In vivo single-cargo control validation and tumor immune activation induced by mBAP1/siENO1@LNP-OTP.**

A: Representative bioluminescence images of orthotopic K7M2-luciferase tumor-bearing mice treated with siENO1@LNP-OTP or mBAP1@LNP-OTP at Days 8, 12, and 16.

B: Quantification of bioluminescence intensity in PBS, siENO1@LNP-OTP, mBAP1@LNP-OTP, and mBAP1/siENO1@LNP-OTP groups at the indicated time points.

C: Representative images of excised tumors from siENO1@LNP-OTP- and mBAP1@LNP-OTP-treated mice on Day 18.

D: Tumor weight quantification of PBS, siENO1@LNP-OTP, mBAP1@LNP-OTP, and mBAP1/siENO1@LNP-OTP groups on Day 18.

E-F: Relative mRNA expression of immune-related chemokines (Ccl2, Ccl5, Cxcl9, and Cxcl10) and pro-inflammatory cytokines (Il-12, Tnf-α, and Ifn-γ) in tumor tissues from PBS- and mBAP1/siENO1@LNP-OTP-treated mice, determined by qPCR (n=4).

G–I: Representative flow cytometry plots and quantification of CD86+, iNOS+, and TNF-α+ cells within the CD45+CD11b+F4/80+ macrophage population in tumor tissues (n = 4).

J–K: Representative flow cytometry plots and quantification of Granzyme B+ and IFN-γ+ CD8+ T cells in tumor tissues (n = 4).

Data are presented as mean ± s.e.m. Statistical significance was determined by unpaired two-tailed Student’s t-test for two-group comparisons, one-way ANOVA followed by Tukey’s post hoc test for multiple-group comparisons, and two-way ANOVA for time-course analyses. ns, not significant; *P < 0.05, **P < 0.01, ***P < 0.001, ****P < 0.0001.

**Supplement Tables**

**Table S1.** Primer sequences used in the study.

| **Primers** | **Sequence 5’ - 3’** |
| --- | --- |
| iNOS-F | GAGACAGGGAAGTCTGAAGCAC |
| iNOS-R | CCAGCAGTAGTTGCTCCTCTTC |
| IL-1B-F | TGGACCTTCCAGGATGAGGACA |
| IL-1B-R | GTTCATCTCGGAGCCTGTAGTG |
| TNF-a-F | GGTGCCTATGTCTCAGCCTCTT |
| TNF-a-R | GCCATAGAACTGATGAGAGGGAG |
| IL-6-F | TACCACTTCACAAGTCGGAGGC |
| IL-6-R | CTGCAAGTGCATCATCGTTGTTC |
| Arg2-F | CACCTCTCACCACTGTATCTGG |
| Arg2-R | CCAGGAAAATCCTGGCAGTTGTG |
| CXCL10-F | ATCATCCCTGCGAGCCTATCCT |
| CXCL10-R | GACCTTTTTTGGCTAAACGCTTTC |
| IL-10-F | CGGGAAGACAATAACTGCACCC |
| IL-10-R | CGGTTAGCAGTATGTTGTCCAGC |
| Arg 1-F | CATTGGCTTGCGAGACGTAGAC |
| Arg 1-R | GCTGAAGGTCTCTTCCATCACC |
| Actin-F | CATTGCTGACAGGATGCAGAAGG |
| Actin-R | TGCTGGAAGGTGGACAGTGAGG |
| GAPDH-F | CATCACTGCCACCCAGAAGACTG |
| GAPDH-R | ATGCCAGTGAGCTTCCCGTTCAG |
| Bap1-F | CACCAGTGGTGGGTGCATCAAG |
| Bap1-R | GTGCTTAGGCACCTCTGAACTC |
| Gclm-F | CAGAAGGATCACCAGTAGTCGG |
| Gclm-R | ATAGCCTCCTCGACCCACTTCT |
| Slc40a1-F | GCTCCAAGAGTTCTCACTGTGAC |
| Slc40a1-R | GTTTTTCTCGCCAAAGCTGTGGC |
| Gsta1-F | CTCTGAGGAAGA ACTGAAGCCAC |
| Gsta1-R | TGCGATCTGGTTCTCCTTCAGC |
| Gsta3-F | GCTACAGGAGAATCACAAGCAGC |
| Gsta3-R | ACGTCTTATCCAAGTGGTTTATGG |
| Tfrc-F | GAAGTCCAGTGTGGGAACAGGT |
| Tfrc-R | CAACCACTCAGTGGCACCAACA |
| Slc7a11-F | CTTTGTTGCCCTCTCCTGCTTC |
| Slc7a11-R | CAGAGGAGTGTGCTTGTGGACA |
| Gpx4-F | CCTCTGCTGCAAGAGCCTCCC |
| Gpx4-R | CTTATCCAGGCAGACCATGTGC |
| Ccl2-F | GCTACAAGAGGATCACCAGCAG |
| Ccl2-R | GTCTGGACCCATTCCTTCTTGG |
| Cxcl9-F | CCTAGTGATAAGGAATGCACGATG |
| Cxcl9-R | CTAGGCAGGTTTGATCTCCGTTC |
| Cxcl10-F | ATCATCCCTGCGAGCCTATCCT |
| Cxcl10-R | GACCTTTTTTGGCTAAACGCTTTC |
| Ifng-F | CAGCAACAGCAAGGCGAAAAAGG |
| Ifng-R | TTTCCGCTTCCTGAGGCTGGAT |
| Il12b-F | TTGAACTGGCGTTGGAAGCACG |
| Il12b-R | CCACCTGTGAGTTCTTCAAAGGC |
| Ccl5-F | CCTGCTGCTTTGCCTACCTCTC |
| Ccl5-R | ACACACTTGGCGGTTCCTTCGA |
| Tnf-F | GGTGCCTATGTCTCAGCCTCTT |
| Tnf-R | GCCATAGAACTGATGAGAGGGAG |

**Table S2.** siRNA sequences used in the study.

| **Primers** | **Sense Sequence 5’-3** | **Antisense** |
| --- | --- | --- |
| siENO1-1 | GCGUCUCCAGACCCAUUAA(dT)(dT) | UUAAUGGGUCUGGAGACGC |
| siENO1-2 | CGAGAUGGAUGGAACAGAA(dT)(dT) | UUCUGUUCCAUCCAUCUCG |
| siENO1-3 | CAGUGGUGUCUAUCGAAGA(dT)(dT) | UCUUCGAUAGACACCACUG |
| shENO1 | 5′-CCGGGCTTCTGTTCGACCTGACAAACTCGAGTTTGTCAGGTCGAACAGAAGCTTTTTG-3′ |  |
| shNC | 5′-CCGGCCTAAGGTTAAGTCGCCCTCGCTCGAGCGAGGGCGACTTAACCTTAGGTTTTTG-3′ |  |

**Table S3. Antibodies used in the study.**

| **Antibody** | **Catalog#** | **Manufacturer** |
| --- | --- | --- |
| ENO1 | ET1705-56 | HUABIO |
| GAPDH | 10494-1-AP | Proteintech |
| β-Actin | 66009-1-Ig | Proteintech |
| E-Cadherin | 0407-25 | HUABIO |
| N-Cadherin | ET1607-37 | HUABIO |
| Vimentin | ET1610-39 | HUABIO |
| CD9 | ET1601-9 | HUABIO |
| Akt | 9272 | Cell Signaling Technology |
| p-Akt(Ser473) | 4060 | Cell Signaling Technology |
| p-Akt(Thr308) | 13038 | Cell Signaling Technology |
| GPX4 | ET1706-45 | HUABIO |
| SLC7A11(xCt) | HA600098 | HUABIO |
| DYKDDDDK Tag ( FLAG ) | HA601167 | HUABIO |
| Arg1 | ET1605-8 | HUABIO |
| TSG101 | # ET1701-59 | HUABIO |
| Calnexin | # ET1611-86 | HUABIO |
| IFN-γ | 113605 | Biolegend |
| Granzyme B (GzmB) | 168803 | Biolegend |
| TNF-α | 506303 | Biolegend |
| BAP1 | # HA724303 | Biolegend |

**Table S4.** Clinicopathological characteristics of osteosarcoma patients included in the ENO1 immunohistochemical analysis

| Characteristic |  | Overall |
| --- | --- | --- |
| Age at diagnosis (years) | ≥20 | 61 |
|  | ＜20 | 123 |
| Sex | Male | 91 |
|  | Female | 93 |
| Tumor location | Femur | 117 |
|  | Tibia | 35 |
|  | Humerus | 14 |
|  | Ulna | 5 |
|  | Fibula | 3 |
|  | Radius | 4 |
|  | Others | 6 |
| Metastasis | Present | 98 |
|  | Absent | 86 |
| Chemotherapy regimen | MAP | 171 |
|  | MAPI | 13 |
| Histological subtype | Conventional osteosarcoma | 131 |
|  | -Osteoblastic | 103 |
|  | -Chondroblastic | 25 |
|  | -Fibroblastic | 3 |
|  | Telangiectatic | 11 |
|  | Parosteal | 12 |
|  | Others | 3 |
|  | Unknown | 27 |
